# Hypoxia leads to decreased autophosphorylation of the MET receptor but promotes its resistance to tyrosine kinase inhibitors

**DOI:** 10.18632/oncotarget.25472

**Published:** 2018-06-05

**Authors:** Meriem Sarah Mekki, Alexandra Mougel, Audrey Vinchent, Charlotte Paquet, Marie-Christine Copin, Catherine Leroy, Zoulika Kherrouche, Jean-Paul Bonte, Oleg Melnyk, Jérôme Vicogne, David Tulasne

**Affiliations:** ^1^ University Lille, CNRS, Institut Pasteur de Lille, UMR 8161 - M3T – Mechanisms of Tumorigenesis and Target Therapies, F-59000 Lille, France; ^2^ University of Lille, CNRS, Inserm, CHU Lille, Institut Pasteur de Lille, U1019 - UMR 8204 - CIIL -Centre d'Infection et d'Immunité de Lille, F-59000 Lille, France; ^3^ University Lille, Institut de Pathologie, CHU Lille, Avenue Oscar Lambret, F-59000 Lille, France; ^4^ EA 4481 Faculté des Sciences Pharmaceutiques et Biologiques, Université de Lille, 59006 Lille, France

**Keywords:** receptor tyrosine kinase, hepatocyte growth factor/scatter factor, MET, hypoxia, tyrosine kinase inhibitor

## Abstract

The receptor tyrosine kinase MET and its ligand, the Hepatocyte Growth Factor/Scattor Factor (HGF/SF), are essential to the migration, morphogenesis, and survival of epithelial cells. In addition, dysregulation of MET signaling has been shown to promote tumor progression and invasion in many cancers. Therefore, HGF/SF and MET are major targets for chemotherapies. Improvement of targeted therapies requires a perfect understanding of tumor microenvironment that strongly modifies half-life, bio-accessibility and thus, efficacy of treatments. In particular, hypoxia is a crucial microenvironmental phenomenon promoting invasion and resistance to treatments.

Under hypoxia, MET auto-phosphorylation resulting from ligand stimulation or from receptor overexpression is drastically decreased within minutes of oxygen deprivation but is quickly reversible upon return to normoxia. Besides a decreased phosphorylation of its proximal adaptor GAB1 under hypoxia, activation of the downstream kinases Erk and Akt is maintained, while still being dependent on MET receptor. Consistently, several cellular responses induced by HGF/SF, including motility, morphogenesis, and survival are effectively induced under hypoxia. Interestingly, using a semi-synthetic ligand, we show that HGF/SF binding to MET is strongly impaired during hypoxia but can be quickly restored upon reoxygenation. Finally, we show that two MET-targeting tyrosine kinase inhibitors (TKIs) are less efficient on MET signalling under hypoxia. Like MET loss of phosphorylation, this hypoxia-induced resistance to TKIs is reversible under normoxia. Thus, although hypoxia does not affect downstream signaling or cellular responses induced by MET, it causes immediate resistance to TKIs. These results may prove useful when designing and evaluation of MET-targeted therapies against cancer.

## INTRODUCTION

MET is the high-affinity receptor tyrosine kinase (RTK) for the Hepatocyte Growth Factor/Scatter Factor (HGF/SF). The *MET* gene was identified as an oncogene in tumorigenicity assays [1], while HGF/SF was discovered independently as a growth factor for hepatocytes [2] and as a scatter factor for epithelial cells [3, 4]. The MET receptor is expressed mainly at the surface of cells from epithelial origin, whereas HGF/SF is mostly secreted by fibroblasts. This ligand-receptor pair plays a crucial role in the epithelial-mesenchymal dialogue during embryonic development and later during tissue regeneration and homeostasis in adults [5].

The MET receptor is a ∼190-kDa glycoprotein comprising an N-terminal HGF/SF-binding extracellular domain, a single transmembrane domain, and an intracellular domain containing the kinase and C-terminal domains [6]. HGF/SF binding to MET triggers its dimerization and its activation by *trans*-autophosphorylation of two tyrosine residues (Y1234 and Y1235) located within the kinase domain. Thereafter, phosphorylation of tyrosines Y1349 and Y1356, located into the MET C-terminal domain and constituting a multisubstrate docking site, is sufficient to promote signal transduction and the biological functions of MET. Signaling proceeds through recruitment of an array of signaling mediators such as PhosphoInositide 3-Kinase (PI3K) and the c-Casitas B-lineage Lymphoma (c-CBL) protein and adaptors such as Growth Factor Receptor–Bound protein 2 (GRB2) and GRB2-Associated-Binding protein 1 (GAB1) [7–10]. GAB1 is a crucial effector recruited by MET. Its phosphorylation creates multiple binding sites for signaling proteins such as the Shp-2 tyrosine phosphatase and the p85 subunit of PI3K [9–11]. Hence, ligand-dependent activation of MET leads to activation of multiple downstream signaling pathways such as the PI3K/Akt and RAS/Erk pathways.

Many mechanisms leading to MET inactivation have been brought to light. First, ligand-dependent phosphorylation of the juxtamembrane tyrosine 1003 triggers recruitment of the E3 ubiquitin ligase c-CBL and receptor internalization and degradation [12]. MET also undergoes proteolytic cleavages by endopeptidases which regulate its half-life under steady-state conditions [13].

HGF/SF-MET signaling is involved in a wide diversity of cellular responses, such as colony dispersal (scattering), proliferation, epithelial cell motility, branched tubule formation upon culture on matrix substitutes, and cell survival upon induction of apoptosis [9, 11, 14–17]. In agreement with the biological roles of HGF/SF-MET signaling, its dysregulation is directly linked to tumorigenesis. In many tumors MET, HGF/SF, or both are overexpressed and associated with poor prognosis [14, 18–22]. MET mutations have also been linked to cancer in patients suffering from hereditary papillary renal carcinoma (RCC) [23, 24]. Sporadic mutations have been detected in many cancer types, including RCC and non-small-cell lung cancers (NSCLC) [25]. In cells displaying *met* gene amplification, ligand-independent activation of the receptor can also occur. This is the case, for example, in about 5% of gastric cancers [26].

Many MET-targeting therapies are under growing development, as attested by more than 300 clinical trials [27]. The investigated molecules include tyrosine kinase inhibitors (TKIs) and antibodies interfering with the ligand/receptor interaction. Most TKIs are small molecules specifically targeting the MET kinase domain, such as ATP-competitive inhibitors [28]. Recently, potential mechanisms of acquired resistance to MET-targeting therapies have been described. In gastric carcinoma cell lines, resistance to MET-targeting TKIs can result either from a point mutation in the MET activation loop or from EGFR activation bypassing inhibition of MET downstream signaling [29]. Another study focusing on gastric cancer and NSCLC models has established *met* amplification followed by *KRAS* amplification [30].

Besides genetic changes leading to alterations and dysregulations of signal transduction pathways, the microenvironment plays an important role in tumor establishment, progression, spread, and metastasis [31, 32]. In particular, when high cell metabolism and rapid proliferation cause a solid tumor to outgrow its blood supply, tumor cells are exposed to conditions (hypoxia and nutrient deficiency) that trigger major changes in their physiology [33–35]. During hypoxia, the oxygen pressure to which tumor cells are exposed can fall below 1% [31]. Cell adaptation to hypoxia is mediated mainly by activation of transcription factors of the hypoxia-inducible factor (HIF) family. This response is regulated post-transcriptionally through stabilization of the oxygen-labile alpha subunit of HIF [36, 37]. Under normoxia, HIF1a is hydroxylated on several proline residues, in an oxygen-dependent reaction [38], by a family of prolyl hydroxylases (PHDs). Hydroxylated HIF1a is recognized by the von Hippel-Lindau (VHL) tumor suppressor ubiquitin ligase and hence ubiquitinylated and degraded. Under hypoxic conditions, HIF1a is stabilized and dimerizes with nuclear HIF1b. This complex binds to hypoxia-responsive elements in DNA and enhances transcription of target genes involved in promoting adaptations to hypoxia.

In patients, hypoxia is viewed as a marker of poor prognosis, associated with uncontrolled tumor growth, angiogenesis, invasiveness, metastasis, and resistance to radio- and chemotherapy [32, 39]. For instance, it promotes angiogenesis through upregulation of VEGF and VEGFR2 synthesis, which may further enhance metastatic spread and promote intravasation [40–42]. Hypoxia additionally enhances receptor-tyrosine-kinase-mediated signaling [43], increasing *hgf/sf* and *met* expression [44, 45]. In response to HGF/SF, the downstream RAS-Erk pathway is activated and invasion increases [45–47].

Here we have examined how hypoxia affects MET receptor activation. We show that it strongly and dynamically decreases the level of MET tyrosine phosphorylation, surprisingly without affecting downstream signaling pathways and biological responses. Importantly, we show that hypoxia reduces MET sensitivity to TKIs. This work highlights a novel mechanism of immediate cell resistance to TKIs under hypoxic conditions.

## RESULTS

### Hypoxia causes a drastic, dynamic, reversible decrease in MET phosphorylation

**Figure 1:**
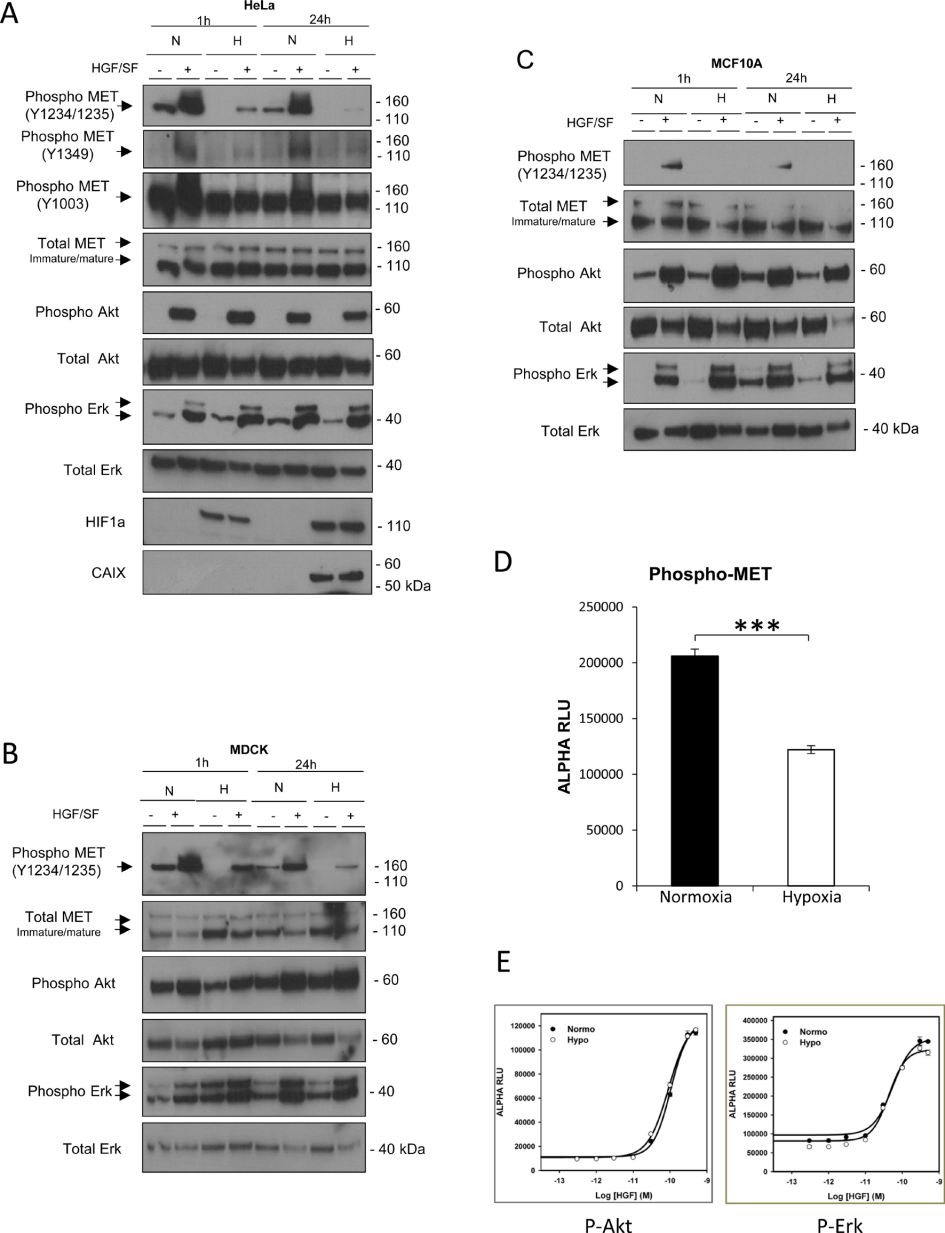
Effect of hypoxia on MET phosphorylation and activation of the Akt and Erk downstream pathways Hela (**A**), MDCK (**B**), and MCF10A (**C**) cells were incubated for 1 or 24 h under normoxic or hypoxic conditions and then treated or not for 10 minutes with 10 ng/mL HGF/SF. For each cell line, the same amount of protein was analyzed by western blotting with antibodies directed against the indicated phosphorylated residue(s) in the MET kinase domain, juxtamembrane domain, or C-terminal domain or against one of the following: the MET kinase domain, phosphorylated Akt, Akt, phosphorylated Erk, Erk2, or the hypoxia marker HIF1a or carbonic anhydrase IX (CAIX). The positions of prestained molecular weight markers are indicated. Arrows indicate the positions of precursor and mature full-length MET and Erk1/2 proteins. (**D**) GTL16 cells were placed under normoxic or hypoxic conditions for 1.5 h. Cell lysates were incubated for AlphaScreen specific phospho-MET quantitation. Error bars represent standard deviations (± SD). (**E**) MCF10A cells were placed under normoxic or hypoxic conditions for 1.5 h, then treated for 10 minutes with HGF/SF at 50, 30, 10, 3, 1, 0.3, or 0 ng/mL. Cell lysates were incubated for AlphaScreen specific phospho-Erk and phospho-Akt quantitation. Error bars represent standard deviations (± SD).

To investigate possible modulatory effects of hypoxia on MET signaling, we incubated various cell lines for 1 h or 24 h under normoxia (21% O_2_) and hypoxia (1% O_2_). Hypoxia was assessed on the basis of HIF1a stabilization or expression of carbonic anhydrase IX (CAIX). Under normoxia, as expected, HGF/SF treatment of HeLa human cervix adenocarcinoma cells induced substantial phosphorylation of MET tyrosine residues Y1234/1235 and led to activation of the Akt and Erk kinases, as revealed by detection of their phosphorylated forms. In contrast, hypoxia caused a strong reduction in MET phosphorylation (Figure 1A), not only at residues Y1234/1235, located in the kinase domain, but also at tyrosines Y1003, into the juxtamembrane domain, and Y1349, into the C-terminal domain. Similar results were obtained with various normal epithelial cell lines, including the canine kidney cell line MDCK (Figure 1B) and the human mammary fibrocystic disease cell line MCF10A (Figure 1C). Quantification of phosphorylated tyrosines Y1234/1235 by AlphaScreen^®^ SureFire technology in ligand independent MET-activated GTL16 cells revealed a 41% phosphorylation decrease under hypoxia, as shown in Figure 1D. A 90% decrease in MET phosphorylation is observed in MCF10A cells by quantification of the chemiluminescence signal (Supplementary Figure 1D). Despite this decrease, activation of the downstream signaling kinases Erk and Akt seemed unaffected (Figure 1A–1C). When AlphaScreen technology was used to obtain quantitative dose-response curves for Akt and Erk activation, no significant difference was measured between normoxic and hypoxic conditions in MCF10A cells (Figure 1E). Similar results were obtained with cell lines established from human tumors, including the human fibrosarcoma cell line HT1080 (Supplementary Figure 1A), the human hepatocarcinoma cell line HepG2 (Supplementary Figure 1B) or with primary adult epidermal keratinocytes (Supplementary Figure 1C). In all these cell types, MET phosphorylation was low or below the detection level under hypoxia, while downstream activation of Akt and Erk was unchanged or even stronger. This suggests that the observed effect of hypoxia is widespread and not cell type specific. While hypoxia led to extremely reduced MET phosphorylation, EGF-induced EGFR phosphorylation was also affected in MCF10A but in a much lesser extent than for MET (Supplementary Figure 1E). It is worth stressing that we observed no major difference between hypoxia and normoxia in the global tyrosine phosphorylation pattern (as detected with a global anti-phosphotyrosine antibody), apart from a band of about 170 kDa that might correspond to activated MET (Supplementary Figure 1F).

**Figure 2:**
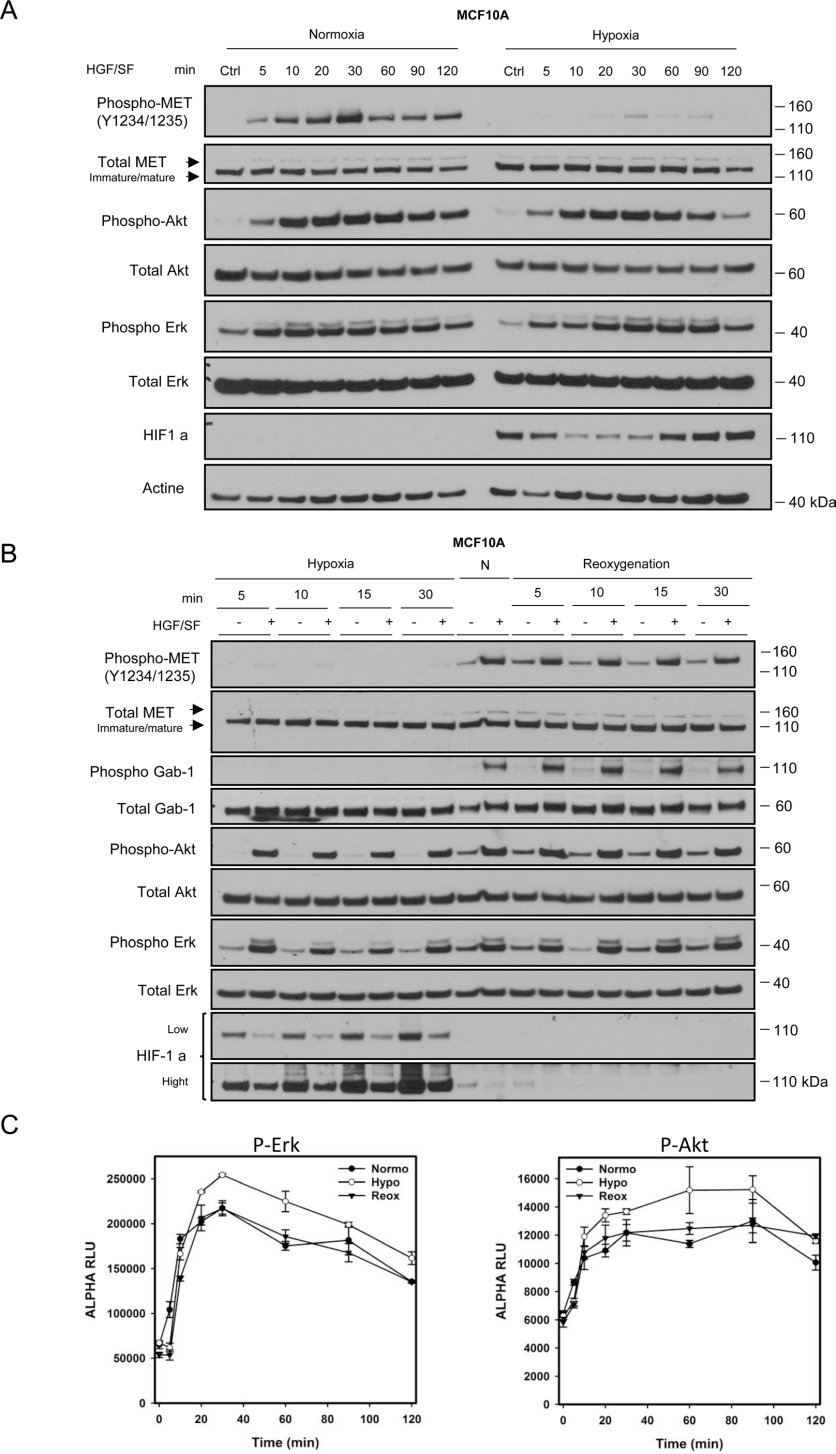
Dynamics of the hypoxia-triggered decrease in MET phosphorylation and its reversal upon reoxygenation (**A**) MCF10A cells were incubated under normoxia or hypoxia for 1 h. They were then treated, under the same oxygen pressure, with 10 ng/mL HGF/SF for 5, 10, 20, 30, 60, 90 and 120 minutes. A control (Ctrl) without any HGF/SF stimulation was also performed. The same amount of protein was analyzed by western blotting with antibodies directed against: phosphorylated residues in the MET kinase domain, the MET kinase domain, the hypoxia marker HIF1a, phosphorylated Akt, Akt, Erk2, phosphorylated Erk, and actine. The positions of prestained molecular weight markers are indicated. Arrows indicate the positions of precursor and mature full-length MET. (**B**) MCF10A cells were incubated under hypoxia for 5, 10, 15 or 30 minutes. Another set of cells were incubated under hypoxia for 1 h and then returned to normoxia for 5, 10, 15 or 30 min (re-oxygenation). A control under normoxic (N) conditions was also included. The same amount of protein was analyzed by western blotting as previously described with the addition of GAB1 and its phosphorylated form. (**C**) MCF10A cells were placed under normoxic or hypoxic conditions for 1 h or hypoxia for 1 h and then normoxia for 10 minutes (reoxygenation). Cells were then treated at the indicated time with 10 ng/mL of HGF/SF. Cell lysates were incubated for AlphaScreen specific phospho-Erk and phospho-Akt quantitation. Error bars represent standard deviations (*n =* 3; ± SD).

To evaluate whether the reduced MET phosphorylation observed after 10 minutes of HGF/SF treatment might merely reflect delayed MET stimulation under hypoxia, time courses of HGF/SF signaling were established for MCF10A cells. Under normoxia, the level of phosphorylated MET displayed a bell-shaped curve, with a maximum at 20–30 min. Under hypoxia, the signal for phosphorylated MET was much lower at every time point, while Akt and Erk phosphorylation still occurred and were not delayed (Figure 2A). Clearly, the reduced MET phosphorylation observed at 10 min under hypoxia did not reflect a delayed response. Interestingly, MET activation under hypoxia led to a reduce level of HIF1a between 10 and 30 min, i.e. during the maximum activation of MET.

Next, MCF10A cells were incubated under hypoxic conditions for 5 to 30 minutes in order to analyze how the MET phosphorylation level varies over time. This experiment confirmed that the strong decrease in MET phosphorylation under hypoxia is extremely quick. Importantly, MET phosphorylation was restored also within 5 minutes when the cells were reoxygenated (Figure 2B). To assess the maintain without delay of Erk and Akt activations under the different culture conditions, their phosphorylation were quantified using AlphaScreen technology upon HGF time course stimulations under hypoxia, normoxia and reoxygenation. Under normoxia, the level of Erk and Akt phosphorylation displayed the expected bell-shaped curve, with a maximum at 20–30 min for Erk and 20–90 min for Akt. Under hypoxia, Akt and Erk phosphorylation conserved similar bell-shaped curve without any delay, with however a slight increase Erk and Akt phosphorylation at all the time course points. After reoxygenation, Erk and Akt time course activations are strictly similar to the initial normoxic condition (Figure 2C). Taken together, these results show that reduction of MET phosphorylation during hypoxia is a fast, dynamic and reversible phenomenon.

### Under hypoxia, activation of the Akt and Erk pathways in response to HGF/SF depends on MET and GAB1

**Figure 3:**
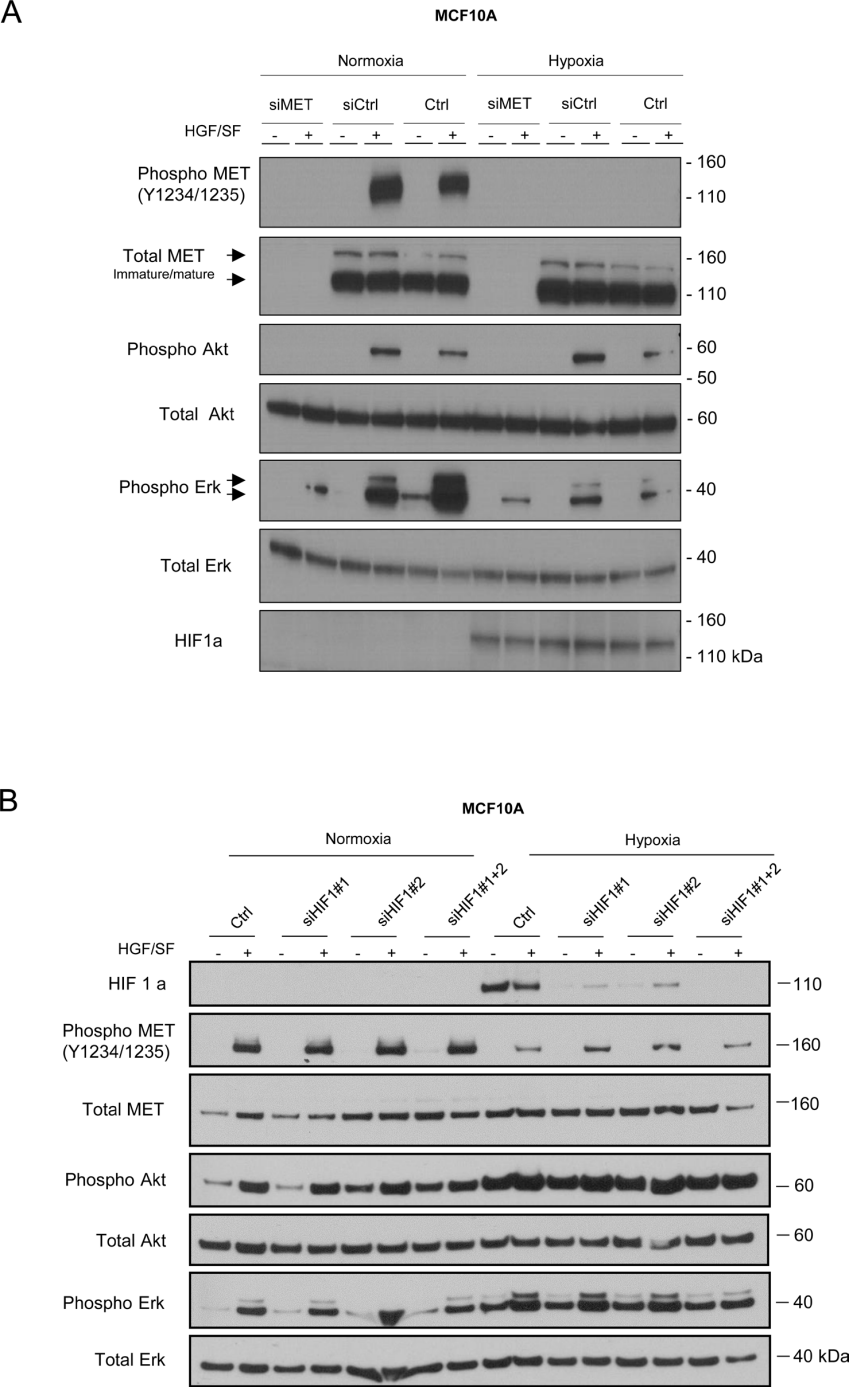
Involvement of MET in Akt and Erk pathway activation under hypoxia MCF10A cells were transfected with a pool of three MET-targeting siRNAs (20 nM) or a control siRNA (siCtrl). A control without siRNA was also included (Ctrl) (**A**). MCF10A cells were transfected with two HIF1a-targeting siRNAs (20 nM), independently or together, or a control siRNA (siCtrl) (**B**). The cells were then placed for 1 h under normoxic or hypoxic conditions and treated or not for 10 min with 10 ng/mL HGF/SF. In each experiment, the same amount of protein was analyzed by western blotting with antibodies directed against: phosphorylated residues in the MET kinase domain, the MET kinase domain, phosphorylated Akt, Akt, phosphorylated Erk, Erk2, phosphorylated GAB1, GAB1, or hypoxia marker HIF1a. The positions of prestained molecular weight markers are indicated. Arrows indicate the positions of precursor and mature full-length MET and Erk1/2 proteins.

In order to assess whether Akt and Erk phosphorylation still depend on MET and GAB1 activation in cells cultured under hypoxia, endogenous expression of the MET and GAB1 proteins was inhibited with specific siRNAs. MCF10A cells were transfected with a pool of siRNAs targeting MET (Figure 3A) or GAB1 (Supplementary Figure 2). MET or GAB1 knockdown was found to inhibit Akt phosphorylation under both normoxia and hypoxia. It also inhibited Erk phosphorylation, albeit to a lesser extent. Thus, although MET phosphorylation is strongly reduced under hypoxia, the HGF/SF-induced downstream Akt and Erk signaling pathways remain directly dependent on the MET receptor and the GAB1 adaptor functionality.

### Hypoxia-inducible factor stabilization does not decrease MET phosphorylation

To investigate involvement of HIF1a stabilization in the decrease of MET phosphorylation, HIF1a was silenced by specific siRNAs. Silencing of HIF1a did not affect decrease MET phosphorylation induced by HGF observed under hypoxic condition (Figure 3B). Furthermore, we performed HGF stimulation in Caki-2 cells expressing dominant negative truncated form of von Hippel-Lindau (VHL), the E3 ubiquitin ligase of HIF1a. While, as expected, HIF1a was already stabilized in normoxia, HGF stimulation induced efficient MET phosphorylation in normoxia, which decreased in hypoxia (Supplementary Figure 3A).

Previous studies have shown MET phosphorylation to be mostly unaffected by the presence of cobalt chloride (CoCl_2_), which is often used to mimic hypoxia because it promotes HIF1a stabilization [47]. While both CoCl_2_ and hydralazine were found to stabilize HIF1a as expected, they had no effect on the MET phosphorylation level observed upon HGF/SF stimulation. Thus, the effects of these chemical treatments are clearly different from those induced under low oxygen pressure (Supplementary Figure 3B).

To investigate the mechanisms underlying the observed hypoxia-triggered reduction in MET phosphorylation, MCF10A cells were treated with the general tyrosine phosphatase inhibitor sodium orthovanadate. This treatment restored, under hypoxia, levels of MET phosphorylation similar to those observed under normoxia. This suggests that the lower level of MET phosphorylation observed under hypoxia could be due to phosphatase activation (Supplementary Figure 4A). Although the Shp-2 phosphatase is known to regulate MET signaling, treatment with a Shp-2-specific inhibitor failed to restore MET phosphorylation under hypoxia, suggesting the involvement of another phosphatase (Supplementary Figure 4B). Similar results were obtained with a DEP-1-specific inhibitor (data not shown).

### Reduced MET phosphorylation under hypoxia does not alter HGF/SF-induced biological responses

**Figure 4:**
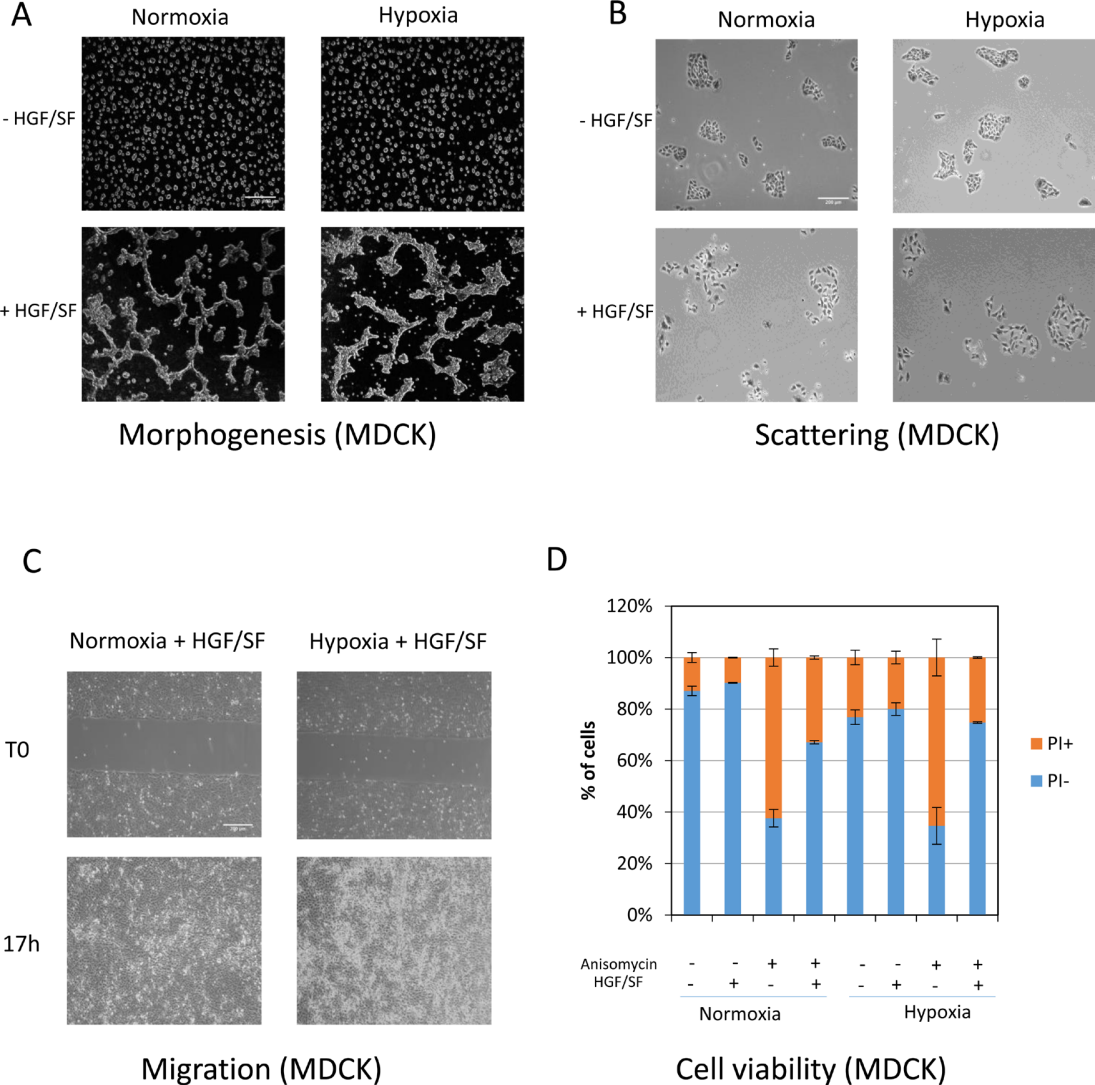
Effect of the MET phosphorylation decrease observed under hypoxia on biological responses induced by HGF/SF (**A**) MDCK cells were seeded on a layer of Matrigel™ for 24 h and then stimulated or not with 10 ng/mL HGF/SF, placed under normoxia or hypoxia for an additional 24 h, and photographed. The white scale bar corresponds to 200 μm. (**B**) MDCK cells were seeded and, after adhesion, serum-starved in the presence or absence of HGF/SF at 10 ng/mL. They were then placed under normoxic or hypoxic conditions for 24 h and fixed, stained with hematoxylin and eosin, and photographed. The white scale bar corresponds to 200 μm. (**C**) MDCK cells were seeded in an IBIDI^®^ insert. The next day, they were treated with 10 µg/mL mitomycin-c to prevent proliferation. An hour later, the mitomycin-C was removed and the cells were left overnight under normoxic or hypoxic conditions. The cells were then photographed. White scale bar: 200 μm. (**D**) MDCK cells were seeded and serum-starved for 24 h after adhesion. The cells were then treated or not with 0.7 µM anisomycin in the presence or absence of 25 ng/mL HGF/SF. The next day, they were stained with propidium iodide (PI) for evaluation of cell viability.

Because hypoxia causes a strong reduction in MET phosphorylation without affecting activation of the two main downstream pathways, we evaluated the impact of oxygen deprivation on various HGF/SF-induced biological responses. Under hypoxia and normoxia, HGF/SF-stimulated MDCK epithelial cells behaved similarly as regards morphogenesis (when cultured on an extracellular matrix substitute), scattering, migration (in gap-closure assays), and survival (when challenged with the apoptosis inducer anisomycin) (Figure 4A–4C, and 4D, respectively). These results show that the drastic decrease in MET phosphorylation observed during hypoxia does not prevent the broad range of biological responses induced by MET in epithelial cells.

### In experimental tumors, hypoxia leads to reduced MET phosphorylation level

**Figure 5:**
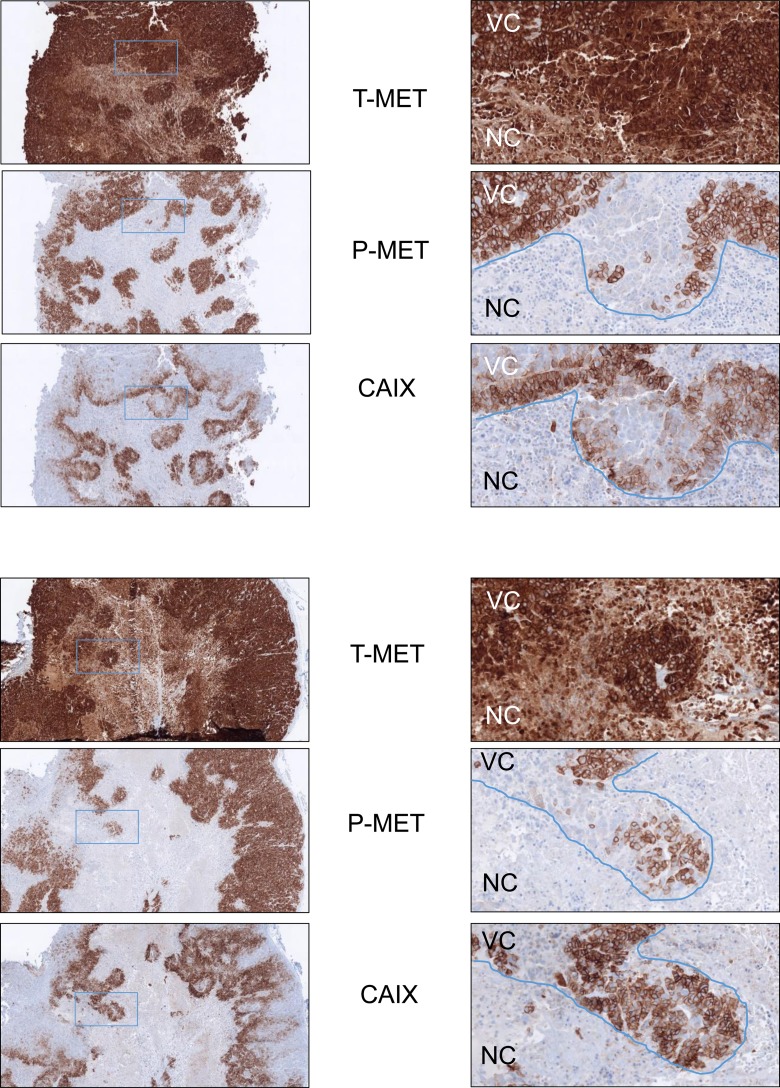
Decreased MET phosphorylation in hypoxic areas of GTL16 tumor xenografts GTL16 cells were xenografted subcutaneously into flanks of SCID mice. Tumors were analyzed by immunohistochemistry on 7-µm-thick sections with antibodies directed against the intracellular domain of MET, the phosphorylated form of MET, or the hypoxia marker CAIX. Two tumors are shown in this figure. Left panels: magnification factor ×4; right panels: magnification factor ×20 (applied to the boxed region). NC: Necrotic tumor cells. VC: Viable tumor cells.

In order to evaluate the MET phosphorylation status *in vivo*, GTL16 tumors were generated in immunodeficient mice. In cell culture, the gastric cancer cell line GTL16 displayed MET overexpression and constitutive phosphorylation, a consequence of the *MET* gene amplification. As observed in other cell lines, the MET phosphorylation level was strongly reduced in GTL16 cells placed under hypoxia for 1 or 24 h and treatment with HGF/SF did not modify MET phosphorylation confirming constitutive MET activation (Supplementary Figure 5). As expected, the tumors displayed intense staining for MET and its phosphorylated form by IHC. The centers of the tumors displayed large necrotic areas surrounded by ribbons of hypoxic cells stained with the hypoxia marker CAIX. Importantly, although most of the hypoxic areas still showed both MET and phospho-MET staining, some of them displayed intense MET staining but no more phospho-MET staining, as shown in two representative samples (Figure 5). Normoxic areas showed no such decoupling between MET and phospho-MET staining. The haematoxylin and eosin staining allowed determination of viable and necrotic areas within the tumor defined by the blue line in Supplementary Figure 6A. Specificity of immunohistochemical staining with the antibody directed against the phosphorylated form of MET was assessed in GTL16 cells treated or not with MET kinase inhibitor PHA-665752 (Supplementary Figure 6B). These *in vivo* data demonstrate that hypoxia generated during tumor growth can lead to reduced MET phosphorylation levels.

### Hypoxia reduced interaction of MET with its ligand

**Figure 6:**
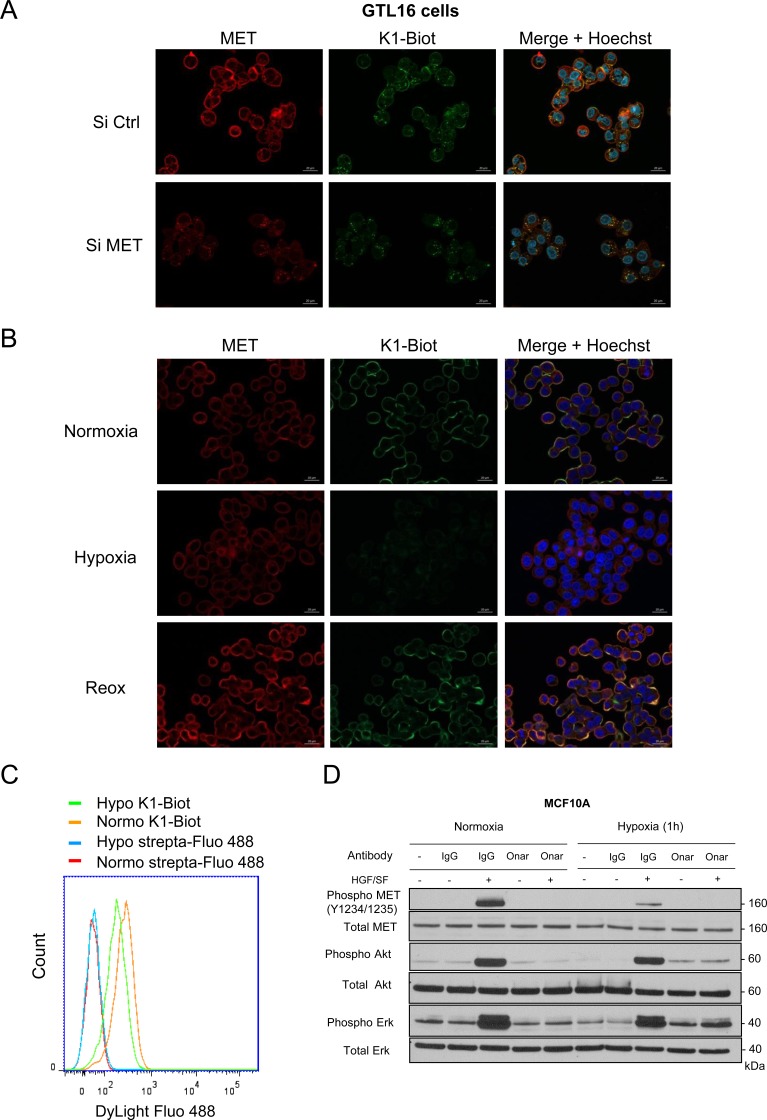
Interaction of K1 subdomain of HGF/SF with MET receptor (**A**) GTL16 cells were transfected with a pool of three MET-targeting siRNAs (90 nM) or a control siRNA (siCtrl) and grown on glass coverslips, (**B**) Cells were placed under normoxic or hypoxic conditions for 2 h. Another set of cells were incubated under hypoxia for 2 h and then returned to normoxia for 2 h (re-oxygenation, Re-ox). (A, B) Cells were incubated 10 min with a complex K1-biotinylated/streptavidin-DyLight 488 (100 nM/50 nM respectively) (green staining, strepta-Fluo 488). Cells were then fixed and labeled with an anti-MET antibody recognizing the intracellular domain of the receptor (red staining) and their nucleus stained with Hoechst (blue staining). An overlay of the three stains is shown (merge) (scale bar = 20 µm). (**C**) Cells were placed under normoxic or hypoxic conditions for 2 h and incubated 10 min with a complex K1-biotinylated/streptavidin-DyLight 488 (100 nM/50 nM respectively) or streptavidin-Dylight 488 alone and analyzed by flow cytometry. (**D**) MCF10A cells were placed under normoxia or hypoxia for 1 h and incubated with 10 μg/ml onartuzumab (Onar) or the corresponding human control IgG (IgG). Cells were then stimulated or not for 10 min with 10 ng/mL HGF/SF. The same amount of protein was resolved by 4–12% Bis-Tris SDS-PAGE and analyzed by western blotting with antibodies directed against phosphorylated residues of the MET kinase domain, the MET kinase domain, phosphorylated Akt, Akt, phosphorylated Erk, or Erk2. The positions of prestained molecular weight markers are indicated.

Because hypoxia induces decrease of MET auto-phosphorylation notably in response to HGF/SF, we proposed that this cellular condition could affect ligand/receptor recognition. In order to assess this hypothesis, we took advantage of the first kringle domain (K1) of HGF/SF, a part of the high affinity HGF binding site for MET, that we produced by total protein chemical synthesis [48, 49]. We notably previously demonstrated that biotinylated K1 associated to streptavidin-sepharose allows the capture of the soluble extracellular domain of MET in conditioned medium [13]. Therefore, we labelled GTL16 cells with the biotinylated-K1 detectable through its association with a fluorophore-conjugated streptavidin. Fluorescent-K1 displayed membrane staining strictly colocalized with those of MET performed with an antibody. Both K1 and anti-MET antibody staining decrease in MET-silenced cells with a pool of siRNA, demonstrating the specificity of the labelling (Figure 6A). Importantly, hypoxia induced a strong decrease of K1 staining, while anti-MET antibody staining remained unchanged. Similar drastic decrease of K1 staining was also observed in EBC1 cells cultured under hypoxia (Supplementary Figure 7). Reoxygenation restored K1 labelling, demonstrating the reversibility of this process (Figure 6B). Flow cytometry analysis with the fluorescent K1 confirmed the decrease of the MET labeling under hypoxia (Figure 6C). Taken together, these data indicate that, under hypoxia, association between MET and the high-affinity subdomain of HGF/SF is strongly reduced. This suggests that decrease of MET phosphorylation under hypoxia could be the consequence an alteration of the ligand/receptor recognition.

To assess whether remaining HGF-MET interaction under hypoxia is involved in the efficient activation of the downstream signaling pathways observed in this culture condition, we treated the cells with the monoclonal antibody onartuzumab directed against extracellular domain of MET and able to inhibit HGF-MET interaction [50]. As expected, under normoxia, onartuzumab drastically inhibited HGF-induced MET phosphorylation as well as Erk and Akt phosphorylation compare to treatment with the irrelevant control IgG. Under hypoxia, treatment with the monoclonal antibody inhibited further the already reduced MET phosphorylation induced by HGF. Erk and Akt phosphorylation were also strongly decrease, as efficiently than under normoxia (Figure 6D). This demonstrates that although HGF-MET interaction seems reduced under hypoxia, likely leading to MET phosphorylation decrease, the remaining HGF-MET interaction is still involved in activation of its downstream signaling pathways.

### Hypoxia induces resistance to MET-targeting and EGFR-targeting tyrosine kinase inhibitors

**Figure 7:**
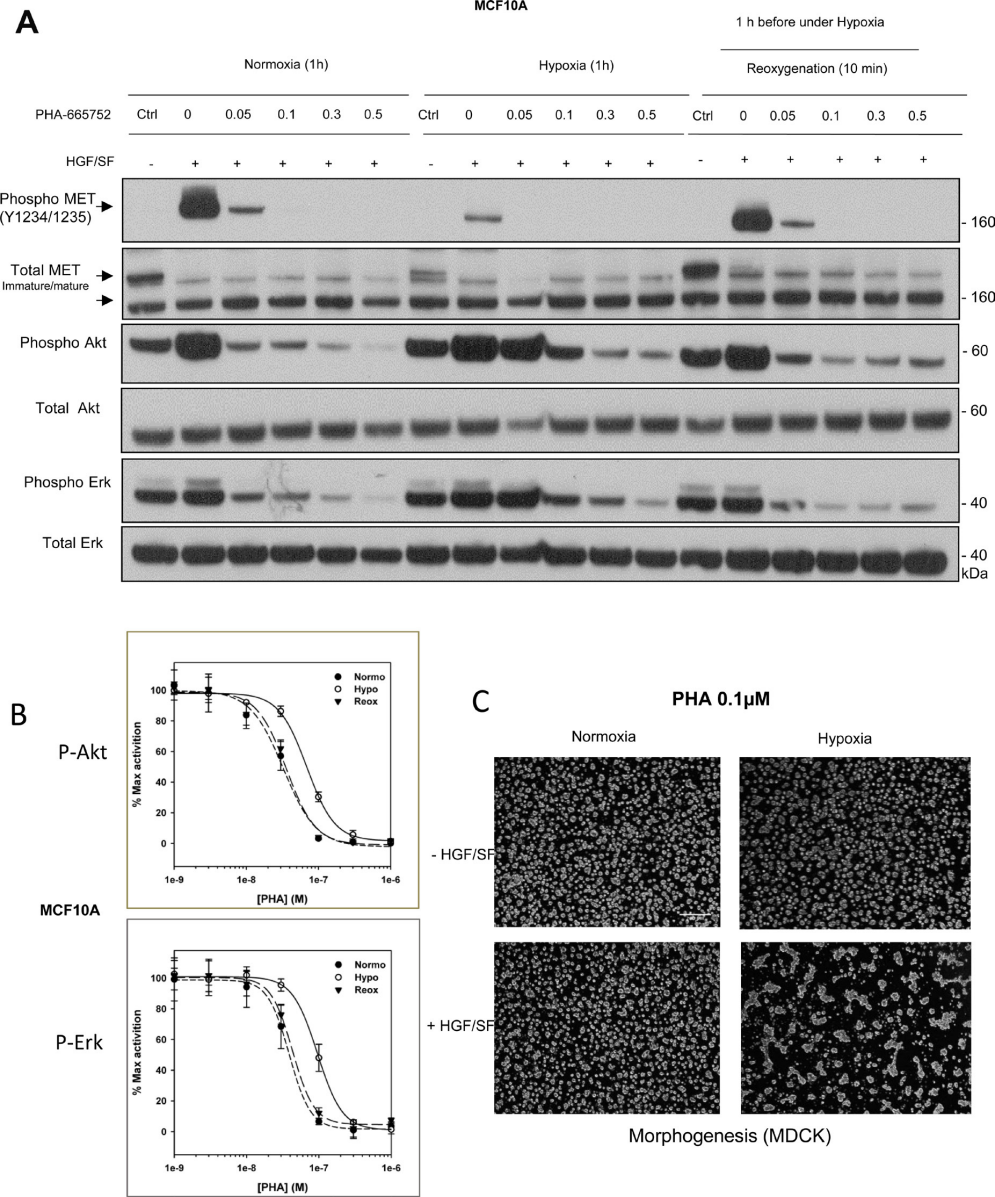
Responses to the tyrosine kinase inhibitor PHA-665752 under hypoxia and reoxygenation (**A**) MCF10A cells were treated with the MET tyrosine kinase inhibitor PHA-665752 at the indicated concentrations and then placed under normoxia or hypoxia for 1 h or under hypoxia for 1 h and then normoxia for 10 minutes (reoxygenation). The cells were then stimulated or not for 10 min with 10 ng/mL HGF/SF. The same amount of protein was resolved by 4–12% Bis-Tris SDS-PAGE and analyzed by western blotting with antibodies directed against phosphorylated residues of the MET kinase domain, the MET kinase domain, phosphorylated Akt, Akt, phosphorylated Erk, or Erk2. The positions of prestained molecular weight markers are indicated. Arrows indicate the positions of precursor and mature full-length MET. (**B**) MCF10A cells were placed under normoxic or hypoxic conditions for 1.5 h or hypoxia for 1.5 h and then normoxia for 10 minutes (reoxygenation) then treated for 10 minutes with HGF/SF 10 ng/mL and PHA-665752 at 10^–3^, 3.10^–3^, 10^–2^, 3.10^–2^, 0.1, 0.3 and 1 µM. A control without HGF/SF and/or PHA-665752 was included. Error bars represent standard deviations (*n =* 3; ± SD). (**C**) MDCK cells were seeded on a layer of Matrigel^™^ for 24 h and then stimulated or not with 10 ng/mL HGF/SF, with or without 0.1 µM PHA-665752. They were then placed under normoxia or hypoxia for an additional 24 h. Finally they were photographed. White scale bar: 200 μm.

Many RTK-targeting therapies rely on the design of ATP mimetics capable of binding to the ATP-binding pocket from the kinase domain. Interestingly, RTKs displaying constitutive kinase activation, and thus an accessible ATP binding site, are more sensitive to such inhibitors. In order to see how hypoxia might affect the sensitivity of the MET and EGF receptors to ATP mimetics, dose-response experiments were performed under hypoxia and normoxia with two MET-targeting TKIs, PHA-665752 and SU11274 and an EGFR-targeting TKI, gefitinib. Figure 7A shows that the concentration of PHA-665752 required to achieve substantial inhibition was higher under hypoxia than under normoxia (0.3 vs. 0.05 µM). When SU11274 was used, it took about 3 times as much inhibitor under hypoxia as under normoxia to achieve a similar level of inhibition (Supplementary Figure 8). Similarly, when ATP mimetic EGFR inhibitor gefitinib was used, it took at least 2 times as much inhibitor under hypoxia as under normoxia to achieve a similar level of inhibition (Supplementary Figure 9). Moreover, PHA-665752 efficiency on HGF/SF-induced Akt and Erk phosphorylations was quantified by AlphaScreen technology. Experiments showed an IC50 2.1 time higher under hypoxia than under normoxia for Akt inhibition and 2.4 higher for Erk inhibition (Figure 7B). Furthermore, treatment with 0.1 μM PHA-665752 did not suppress HGF/SF-induced morphogenesis in MDCK cells cultured under hypoxia, in contrast to the same cells cultured under normoxia (Figure 7C).

As the decrease in MET and EGFR phosphorylation observed under hypoxia was reversible upon reoxygenation, we tested whether sensitivity to ATP mimetics might likewise be restored in cells subjected to the same hypoxia/normoxia sequence. While cells cultured under hypoxia became resistant to both TKI targeting MET (PHA-665752 and SU11274) and targeting EGFR (gefitinib), subsequent restoration of normoxia for 10 min was sufficient to restore sensitivity to these compounds (Figure 7A and 7B and Supplementary Figures 6 and 7). Thus, both the decrease in MET and EGFR phosphorylation and resistance to MET and EGFR-targeting TKIs are highly dynamic and reversible.

## DISCUSSION

The first step in MET activation is trans-autophosphorylation at several crucial tyrosine residues, notably tyrosines 1234 and 1235 within the kinase domain. After this initial step, MET further undergoes autophosphorylation of tyrosines 1349 and 1356 within the C-terminal domain, which constitutes a docking site for numerous substrates involved in downstream signaling. Phosphorylation of tyrosine 1003 is next required for efficient degradation of the ligand-activated receptor and termination of signaling [51]. Here we show that phosphorylation of all these MET residues is drastically reduced, yet not completely inhibited, under hypoxic conditions, while activation of the downstream signaling kinases Akt and Erk remains unaffected. Consequently, the biological responses under the control of MET are still present under hypoxia.

While the MET activation status has already been investigated under hypoxia [47], such a drastic reduction in MET phosphorylation has not been described before. However, it is worth noticing that this decrease of phosphorylation is quickly reversible, a phenomenon only measurable in hypoxia work station in which all steps of the experimental procedure (treatments and cell lysis) are performed under hypoxic condition. For instance, cell treatment and/or lysis outside the hypoxic atmosphere can preclude accurate measure of MET phosphorylation.

Hypoxia has previously been shown to increase *MET* gene expression as a consequence of HIF1a transcription factor stabilization [44, 45, 52]. We have observed no significant increase in the MET mRNA (data not shown) or protein level after 1 to 24 h of hypoxia. Nevertheless, the MET expression level might vary in some cell types or if treatment is prolonged. Therefore, decrease of MET phosphorylation could occur in the early step of hypoxia, a response independent of HIF1a, while long term hypoxia could induce MET expression, a response dependent of HIF1A.

Up to now, MET phosphorylation status under hypoxia has usually been evaluated after treatment with CoCl_2_, a chemical often used to mimic the response of cells to oxygen deprivation. Indeed, CoCl_2_ and hydralazine promote HIF1a stabilization by preventing hydroxylation of some proline residues by oxygen-dependent PHDs. These prolyl-hydroxylations are required to allow HIF1a ubiquitinylation by VHL and to trigger its constitutive degradation by proteasome under normoxic conditions [53–55]. Our work indicates that physical oxygen deprivation strongly reduces MET phosphorylation, in contrast to treatment with CoCl_2_ or hydralazine. Differences between the states induced by low oxygen pressure and by CoCl_2_ treatment have already been described. For instance, oxygen deprivation has been shown to strongly inhibit protein synthesis, while CoCl_2_ has the opposite effect [56, 57].

The fact that hypoxia leads to reduced MET phosphorylation in various cell lines and primary cells tested suggests that this effect is general. In particular, we show that hypoxia does not merely shift MET activation kinetic, since MET phosphorylation remains at a reduced level all along of HGF/SF treatments. Furthermore, modulation of the MET phosphorylation level appears dynamic and reversible, since the level drops after just a few minutes of incubation at low oxygen pressure and rises rapidly to a typical level when the oxygen pressure is restored to normal pressure.

The hypoxia-triggered decrease in MET phosphorylation concerns several tyrosine residues. Notably affected are the adjacent tyrosines 1234 and 1235, required for kinase domain activation. Other tyrosine residues whose phosphorylation relies on MET kinase activity are also less phosphorylated, particularly tyrosine 1003, required for E3 ubiquitin ligase CBL recruitment and subsequent internalization and degradation of the MET receptor. Also, phosphorylation of tyrosine 1349 in the C-terminal docking site is drastically reduced. This effect is accompanied by reduced phosphorylation of the multi-adaptor protein GAB1, known to be recruited to this docking site.

Despite reduced MET and GAB1 phosphorylation, activation of the Akt and Erk downstream signaling kinases is maintained. These results are consistent with those of a previous study demonstrating efficient Erk activation by ligand-stimulated MET under hypoxic conditions [58]. The same study also mentioned, however, a marked decrease in Akt activation we did not observed. We further demonstrate that, even under hypoxia, activation of Akt and Erk still depends on proper MET activation and GAB1 adaptor recruitment. Indeed, MET or GAB1 knockdown with specific siRNAs effectively inhibits HGF/SF-triggered Erk and Akt activation, and phosphorylation of both downstream kinases is markedly inhibited in the presence of a MET-targeting TKI at optimal dosage. A likely explanation of efficient Erk and Akt activation despite strong decrease of MET phosphorylation is that activation of downstream signaling pathways by MET required only a limited activation of the proximal signaling machinery, including MET and GAB1. In this line, we previously demonstrated that effective activation of downstream signaling pathways by MET requires its constitutive degradation by the proteolytic process PS-RIP. Indeed, uncleavable MET receptor displayed an increase of its expression but a decrease its ligand dependent responses [59].

In hypoxic area of experimental tumors in mouse, we evidenced some cells displaying intense MET staining but no more positive staining for phospho-MET, suggesting that decrease of MET phosphorylation under hypoxia occurs also *in vivo*. It is worth noticing that many cells localized in hypoxic area still display MET phosphorylation. However, in cell lines we found that hypoxia induces decrease of MET phosphorylation but does not totally abrogate it, indicating that MET phosphorylation could be still possible in hypoxic cells. In addition, hypoxia is a very dynamic process within the tumor and phosphorylation level of MET is probably quickly modulated depending on oxygen pressure variation. In contrast, CAIX has a transcriptional modulation (HIF1 dependent) that is per nature less dynamic than MET phosphorylation.

HGF/SF-MET signaling induces a broad range of cellular responses, such as morphogenesis, scattering, migration, and cell survival [60, 61]. Consistently with normal activation of downstream signaling events, all these biological responses appear to be maintained under hypoxia. These results suggest that minimal activation of MET and GAB1 under hypoxia is sufficient to promote full responses. This sensitivity might be due to better signal amplification occurring downstream from GAB1 but upstream from Erk and Akt in hypoxic condition.

Phosphatases are well-known downregulators of kinase phosphorylation and activation. Several phosphatases, including Shp-2, DEP-1, and PTP1B, are known to either dephosphorylate MET or regulate its signaling [62–64]. In keeping with a dynamic, reversible modulatory mechanism, we propose that the decreased MET phosphorylation level observed during hypoxia could be the consequence of phosphatase activation. We have accordingly found the general tyrosine phosphatase inhibitor sodium orthovanadate to promote a high level of phosphorylated MET under both normoxic and hypoxic conditions. This supports the view that phosphatase activity plays a major role in regulating the MET phosphorylation status under hypoxia. Previous reports, however, suggest that the activity of the Shp-2 and DEP-1 phosphatases, among other phosphatases including PTP1B and T-cell PTP, is downregulated under hypoxia [65]. The fact that we have found inhibition of Shp-2 and DEP-1 not to restore the MET phosphorylation level suggests that they are not involved in its hypoxia-triggered decrease. It would therefore now be interesting to evaluate the involvement of novel phosphatases whose activity might be increased by hypoxia.

In addition, in order to investigate why MET activation decrease under physical hypoxia while cellular total MET content stay unchanged, we took advantage of the semi-synthetic biotinylated Kringle 1 subdomain of HGF/SF (K1-biot) known to display high affinity for MET [48]. Indeed, we previously demonstrated that K1-biot associates with high affinity to MET, behaves as a potent agonist when dimerized with streptavin [49] and can be use as bait to capture soluble extracellular domain of MET in conditioned medium when complexed with strevidin-sepharose [13]. Here we complexed the K1-Biot with a streptavidin-fluorophore in order to use it as a molecular probe. Indeed, we demonstrated that labelling with K1-Biot/strepta-Fluo displayed a membrane staining strictly colocalized with MET. This labelling disappeared upon MET silencing demonstrating that the semi-synthetic K1-biot can be used as a functional molecular probe to evaluate ligand/receptor interaction. Importantly, we found that under hypoxia labelling of epithelial cells with K1-biot was strongly reduced under hypoxia and was restored after reoxygenation. Therefore, reduction of MET phosphorylation under hypoxia is the likely consequence of a decrease of the ligand/receptor interaction. This altered HGF/SF-MET interaction under hypoxia might involve posttranscriptional modification of MET, since modulation of its phosphorylation according to the oxygen level is dynamic and reversible. Necessity of HGF to bind MET is this mechanism is strongly demonstrated in using onartuzumab, a modified monoclonal antibody that prevent HGF binding without promoting MET dimerization. Treatment by onartuzumab abrogates subsequent MET signaling upon HGF stimulation both in normoxia and hypoxia.

Alternatively, alteration of ligand/receptor interaction could involve accessibility of co-receptor which membrane expression could be modified under hypoxia. Although the molecular mechanism underlying alteration of the HGF/SF-MET interaction has to be identified, this suggests the existence, beside the well-known “outside-in” ligand dependent activation of MET, of an original “inside-out” signal leading to the modification of MET property to bind its ligand. To our knowledge it is the first time that such alteration of HGF/SF-MET interaction is evidenced according to the cellular context and thus could represent a novel molecular mechanism to regulate the MET receptor downstream signaling. Interestingly, recent structural data suggest the existence of two distinct states of MET receptor (open/close conformation) allowing or not ligand binding [66]. It is worth noticing that potential action of phosphatases and decrease of the HGF/SF-MET interaction could synergically contribute to decrease MET phosphorylation observed during hypoxia. Thus, mechanisms regulating MET states during hypoxia must still be elucidated clearly but oxygen pressure could be one of the physical parameter involved.

There is now clear evidence that tumoral hypoxia induces a complex adaptive response leading notably to resistance to therapy. These responses result from a profound genetic reprogramming mainly involving the transcriptional regulator HIF1a. For instance, hypoxia upregulates expression of the multidrug resistance gene MDR1, causing efflux of chemotherapeutic drugs. It can also exert on cells selective pressure for the loss of p53 [67, 68]. Hypoxia-induced resistance to classical chemotherapeutic agents such as cisplatin or doxorubicin also requires transcriptional regulation, involving HIF1a or not [69]. Prior to this work, resistance under hypoxia has not been described as an immediate mechanism, since it appeared to require at least transcriptional regulation.

Numerous strategies for inhibiting RTK activity are under development or already used clinically in cancer treatment. Many TKIs are ATP mimetics which associate with and block the ATP-binding site of the kinase domain. Paradoxically, ATP mimetics are more effective when the kinase catalytic site is in its open conformation, i. e., in its active state [70]. For instance, EGFR-targeting TKIs such as gefitinib are more effective against EGFR variants with activating mutations where direct binding measurements show that gefitinib binds 20 times more tightly to the L858R mutant than to the wild-type enzyme [71]. Regarding the MET receptor, high sensitivity to MET inhibition appears associated with the high phospho-MET levels observed in HGF/SF autocrine cell lines [72].

Reasoning that the strong decrease in phosphorylated MET observed under hypoxia might modify cellular responses to TKIs, we have found that it takes a two- to six-fold-higher dose of the ATP mimetic PHA-665752 or SU-11274 under hypoxia than under normoxia to substantially reduce HGF/SF-triggered phosphorylation of the downstream kinases Erk and Akt. Likewise, although PHA-665752 treatment can abolish cell morphogenesis under normoxia, the same treatment induces only partial inhibition under hypoxia. Interestingly, re-oxygenation restores the response to the inhibitor. This demonstrates that, like the decrease in MET phosphorylation, resistance to MET-targeting TKIs is reversible. Similar resistance to the EGFR TKI gefitinib was found despite weaker decrease of EGFR phosphorylation compared to MET in hypoxia. Nevertheless, it was demonstrated that gefitinib efficiency is highly dependent on its target activation [71].

Recently, several MET-targeting TKIs have been used successfully in clinical trials on patients with MET deregulations such as *met* gene amplification and activating mutations [73–75]. Interestingly, we have evidenced decreased MET phosphorylation under hypoxia in cells displaying these defects. In the same line, EGFR-targeting TKIs are used in clinic to treat lung cancer displaying EGFR mutations. This suggests that resistance to TKIs could occur in the hypoxic areas of tumors harboring these MET and EGFR deregulations, thereby possibly reducing the effectiveness of these agents.

### Experimental procedures

#### Cell culture, cytokines, and drugs

The cell lines HT1080, HS746T, HeLa, HepG2, and GTL16 were maintained in Dulbecco’s Modified Eagle Medium (DMEM, Gibco^®^ Life Technologies^®^) supplemented with 10% fetal bovine serum (FBS, Gibco^®^ Life Technologies^®^). MDCK cells were maintained in DMEM with 1% Non-Essential Amino Acids (NEAA, Life Technologies^®^) and 50 μg/mL biotin (Calbiochem^®^) supplemented with 10% FBS. EBC-1 cells were cultured in Minimum Essential Medium (MEM, Gibco^®^ Life Technologies^®^) supplemented with 10% FBS. MCF10A cells were cultured in Dulbecco’s Modified Eagle Medium/Nutrient Mixture F-12 (DMEM/F12 (1:1), Gibco^®^ Life Technologies^®^) with 20 ng/mL human recombinant Epidermal Growth Factor (Peprotech^®^), 100 ng/mL cholera toxin (Calbiochem^®^), 0.01 mg/mL insulin (Sigma Aldrich^®^), 500 ng/mL hydrocortisone (Calbiochem^®^) supplemented with 5% horse serum (Life Technologies^®^). Caki-2 cells were cultured in RPMI (Gibco^®^ Life Technologies^®^) supplemented with 10% fetal bovine serum. All media were supplemented with 1% Zell Shield™ (Minerva Biolabs^®^). Keratinocytes were maintained in KGM-Gold™ Bullekit™ (Lonza^®^) supplemented with 0.4% BPE (Bovine Pitutary Extract), 0.5 ng/mL hEGF, 5 mg/mL rh Insulin, 100 ng/mL hydrocortisone, GA-1000 (gentamicin, amphotericin B), 1 mM epinephrine, 5 mg/mL transferrin (Lonza^®^).

Cells were cultured in an *InVivo*2 500™ Awel^®^ station in a stable hypoxic environment (1% O_2_) (94% N_2_, 5% CO_2_, 37° C, 75% humidity) or under normoxia (at 37° C in a water-saturated atmosphere containing 5% CO_2_ and 21% O_2_).

Recombinant human HGF/SF was purchased from Peprotech^®^ (Rocky Hill, CT, USA), anisomycin from Santa Cruz^®^, PHA-665752 from Promega^®^ (Madison, WI, USA), SU-11274 from Calbiochem^®^ and gefitinib from Santa Cruz^®^. Mitomycin-c, DMSO, CoCl_2_, hydralazine, and Na_3_VO_4_ were purchased from Sigma^®^ (St Louis, MO, USA).

### Western blotting and antibodies

Cells were cultured in 6-well plates in their respective media and then starved overnight in serum-free medium. They were then lysed with 100 µL RIPA buffer (20 mM HEPES, 1% NP40, 0.1% SDS, 5% glycerol, 142 mM KCl, 5 mM MgCl2, 1 mM EDTA, pH 7.45) supplemented with phosphatase inhibitors (1/200 Phosphatase Inhibitor Cocktail 2 – Sigma^®^ P5726) and protease inhibitors (1/400 Protease Inhibitor Cocktail – Sigma^®^ P1860). For analysis, lysates were centrifuged and the supernatant collected. The total protein concentration was determined with the BCA Protein Assay Reagent Kit (Pierce^®^), and equal amounts of proteins were resolved on NuPAGE 4–12% Bis-Tris gels (Novex^®^ by Life Technologies™). Separated proteins were transferred onto a polyvinyl difluoride membrane in Towbin buffer (10% methanol, 10% Tris-glycine 1X, 0.0025% SDS). The membrane was then equilibrated in blocking buffer (8 g casein/1L, PBS 1X, 0.2% Tween). Proteins were analyzed by western blotting with anti-phosphorylated MET at 1/1000 dilution from stock solution (Tyr 1234/1235, #3077 – Cell Signaling^®^; Tyr 1003, #44-882 Biosource™ Life Technologies^®^; Tyr 1349, #3121 – Cell Signaling^®^), anti-MET (3D4 - Life Technologies^®^, directed against kinase domain, used for WB, “SP44” Spring Bioscience, against C-terminal tail, used for IHC; #3148 Cell Signaling^®^ against C-terminal domain, used for IF, anti-phosphorylated-Akt (Ser473, #9271 - Cell Signaling^®^), anti-Akt (Cell Signaling^®^), anti-phosphorylated-Erk (Thr202/Tyr204, #9106 - Cell Signaling^®^), anti-Erk2 (C14 – Santa Cruz^®^), anti-phosphorylated-GAB1 (Millipore™), anti-GAB1 (#1626-1 - Epitomics^®^), anti-phosphotyrosines (4G10 – Millipore™), anti- HIF1a (Novus biologicals^®^), anti-carbonic anhydrase IX (CAIX – Abcam^®^) and anti-VHL (ab135576 – Abcam^®^). The human monoclonal antibody onartuzumab and its corresponding control IgG were provided by Genentech (CA, USA). After incubation with the appropriate species-specific horseradish-peroxidase-conjugated secondary antibodies (anti-rabbit (#711-035-15), mouse (#115-035-146), or goat (#705-035-003) – Jackson ImmunoResearch Lab^®^), the antigen-antibody complexes were detected with a light-sensitive photographic film (CL-XposureTM Film – Thermo Scientific^®^) after use of an ECL kit (Amercham™ ECL™ Western Blotting Detection Reagents), West Dura Extended Duration Substrate (SuperSignal^®^), or SuperSignal^®^ West Femto Maximum Sensitivity Substrate (ThermoScientific^®^) as required. For quantification of MET phosphorylation, luminescence was captured by digital imaging with a cooled charge coupled device camera (LAS 3000, Fuji, Tokyo, Japan), and quantification was performed with Multigauge V3.0 software. The background-adjusted volume was normalized with respect to an empty well.

### Transfections and RNA interference

Cells were transfected with different siRNAs targeting MET or GAB1 or HIF1a. Three MET-targeting siRNAs or two GAB1-targeting siRNAs or two HIF1a-targeting siRNAs were used as a pool or separately. Transfection was performed according to the reverse transfection protocol. A suspension of 400,000 MCF10A or 250,000 GTL16 cells were incubated for 20 minutes with a mix of 4.5 µL Lipofectamine 2000 (Invitrogen^®^) and 20 nM MET targeting siRNA for MCF10A cells, 90 nM MET targeting siRNA for GTL16 cells or 20 nM GAB1 targeting siRNA. Cells were then plated in a 6-well plate in a final volume of 1.5 mL complete medium. The MET-targeting siRNAs were Stealth siRNAs (Invitrogen^®^) [5′-CCAUUUCAACUGAGUUUGCUGUUAA-3′, 5′-UCCAGAAGAUCAGUUUCCUAAUUCA-3′, and 5′CCGAGGGAAUCAUCAUGAAAGAUUU-3′]. The GAB1-targeting siRNAs were stealth siRNA (Invitrogen^®^) 5′-GAGAGUGGAUUAUGUUGUU-3′ and GAB1 siRNA HSS103902 – chr4 : 144257983 – 144395718). The HIF1a-targeting siRNAs were Stealth siRNAs 5′-CUGAUGACCAGCAACUUGA-3′ and 5′-CCAGCCGCUGGAGACACAAUCAUAU-3′. A negative control Stealth siRNA was also used (Invitrogen^®^). After adhesion, the medium was renewed, first with complete medium and then with serum-free medium 24 hours later. The next day, cells were placed under normoxic or hypoxic conditions for the indicated time and then stimulated or not with 10 ng/mL HGF/SF for 10 minutes.

### Tumor analysis and immunohistochemistry

All experiments with mice were performed according to international ethical guidelines. GTL16 gastric cancer cells in 200 µL PBS were injected subcutaneously (5.10^6^ cells/injection) into the posterior flanks of nine-week-old female SCID mice. After 17 days, the mice were sacrificed. Their tumors were excised and then formaldehyde-fixed (4%) and paraffin-embedded (FFPE). For immunohistochemistry, 7-µm-thick sections were prepared and IHC was performed with antibodies against the intracellular domain of MET (SP44 CONFIRM, Ventana Medical Systems, Roche^®^), the phosphorylated form of MET (Tyr 1234/1235 – #3077/D26 – Cell Signaling^®^), or CAIX (NCL-L-CAIX - Novocastra™) in a Ventana automated instrument (Benchmark XT Ventana instrument). Haematoxylin and eosin staining was performed with a Sakura automated instrument (Tissue-Tek^®^ Prisma^®^). Structure and organization of mice tumors have been determined by an anatomical pathologist.

### Cellular responses

For the migration assay, MDCK cells were seeded (30,000 cells/well) into an IBIDI^®^ insert. The next day, DMEM with 10% FBS was added to the well with 10 µg/mL mitomycin-c to prevent proliferation. Approximately one hour later, cells were photographed and the medium was removed and replaced with DMEM, 10% FBS and the cells were left overnight under normoxic or hypoxic conditions. The next day, the experiment was stopped and the cells were photographed.

For the morphogenesis assay, MDCK cells were seeded (100,000 cells/well) onto a 300 µL layer of Matrigel™ in a 24-well plate and incubated overnight. The next day, 10 ng/mL HGF/SF was added to the medium and the cells were placed under normoxic or hypoxic conditions for another 24 hours. Finally they were photographed.

For the scattering assay, MDCK cells were seeded (2,500 cells/well) into a 12-well plate in medium with 10% FBS. The next day, and after adhesion, the medium was removed and the cells were serum-starved and HGF/SF was added or not (final concentration: 10 ng/mL). The cells were then incubated under normoxic or hypoxic conditions for 24 hours, fixed, stained with hematoxylin and eosin; and photographed.

For the survival assay, MDCK cells were seeded (at 250,000 cells/well) into a 6-well plate. After adhesion, they were serum-starved for 24 hours before addition of DMEM alone or DMEM containing anisomycin (0.7 µM), HGF/SF (25 ng/mL), or both. The next day, the cells were trypsinized and then analyzed with the Tali^®^ Apoptosis Kit (Life Technologies^®^). Briefly, cells were harvested by centrifugation and incubated with Alexa Fluor 488-conjugated annexin V and propidium iodide. The staining intensity was then measured with a Tali^®^ Image-Based Cytometer (Life Technologies^®^) to determine numbers of unstained, singly stained, and doubly stained cells (only data with PI staining are shown).

### Immunofluorescence

Cells were grown on glass coverslips for 24 hours and incubated in normoxia or hypoxic conditions as indicated. Cells were incubated 10 minutes with a K1-biotinylated/streptavidin-DyLight 488 complex (100 nM/50 nM respectively). Then, cells were washed, fixed with paraformaldehyde 4% 10 minutes and permeabilized in PBS-0.5% Triton 10 minutes. The cells were washed and blocked 30 minutes in PBS 0.2% casein. The cells were incubated 1 hour at room temperature with a monoclonal antibody directed against the C-terminal domain of human MET (1/100). The cells were washed with PBS and incubated with Alexa Fluor-conjugated secondary antibodies (Alexa Fluor 594 conjugated anti-mouse IgG diluted to 20 µg/mL). The cells were washed and the nuclei counterstained with Hoechst 33258.

### Flow cytometry

GTL16 and EBC1 cells were incubated 10 minutes with a K1-biotinylated/streptavidin-Dylight 488 complex (100 nM/50 nM respectively) and detached with Accutase^®^ ThermoFisher and wash with PBS. Then cells were fixed with 4% paraformaldehyde for 15 min and washed. The fluorescence intensity of the cells was measured on a Canto II (BD Biosciences). Data were recorded with BD FACSDivaTM software.

### Quantification of phospho-MET, phospho-Akt and phospho-Erk with AlphaScreen^®^

Measurements were performed in 384-well plates according to the manufacturer’s protocols (OptiPlate™-384, PerkinElmer^®^, CA, USA). For the quantification of phospho-MET, phospho-Akt and phospho-Erk, 12,000 cells were seeded into 96-well culture plates for 24 hours and starved overnight. The next day, the plates were placed under normoxic and hypoxic conditions for 1.5 h or under hypoxic conditions for 1.5 h then reoxygenated for 10 minutes, and the cells were treated or not with HGF/SF, PHA-665752, or both at the indicated concentration(s). The cells were then immediately lysed in the manufacturer’s buffer supplemented with phosphatase inhibitors (1/200 Phosphatase Inhibitor Cocktail 2 – Sigma^®^ P5726) and protease inhibitors (1/400 Protease Inhibitor Cocktail – Sigma^®^ P1860). The lysates were transferred to a 384-well microplate and an appropriate reaction buffer added for detection of phosphorylated MET (TGRCMS500), phosphorylated Akt (TGRA4S500) or Erk (TGRES500). The Alpha signal was measured on an EnSpire plate reader, with standard AlphaScreen^®^ settings (PerkinElmer^®^).

## SUPPLEMENTARY MATERIALS FIGURES


