# Supplementary material for: Hypoxia leads to decreased autophosphorylation of the MET receptor but promotes its resistance to tyrosine kinase inhibitors
Source: Oncotarget. 2018 Jun 5;9(43):27039–58. doi: 10.18632/oncotarget.25472 (PMC6007473; doi:10.18632/oncotarget.25472)
Supplement: Supplementary file 1 [file oncotarget-09-27039-s001.pdf]

Hypoxia leads to decreased autophosphorylation of the MET receptor but promotes its resistance to tyrosine kinase inhibitors

SUPPLEMENTARY MATERIALS

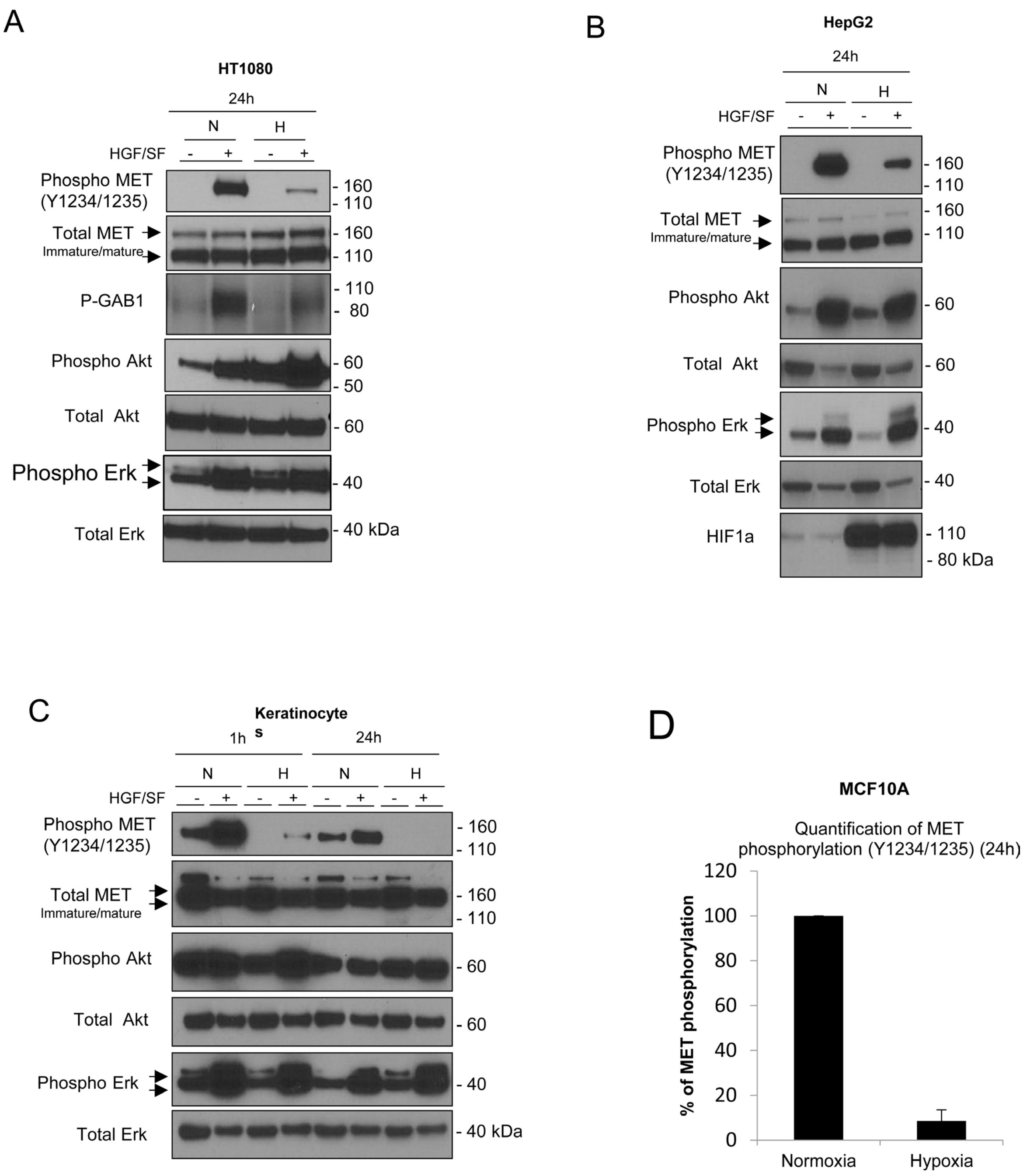

E

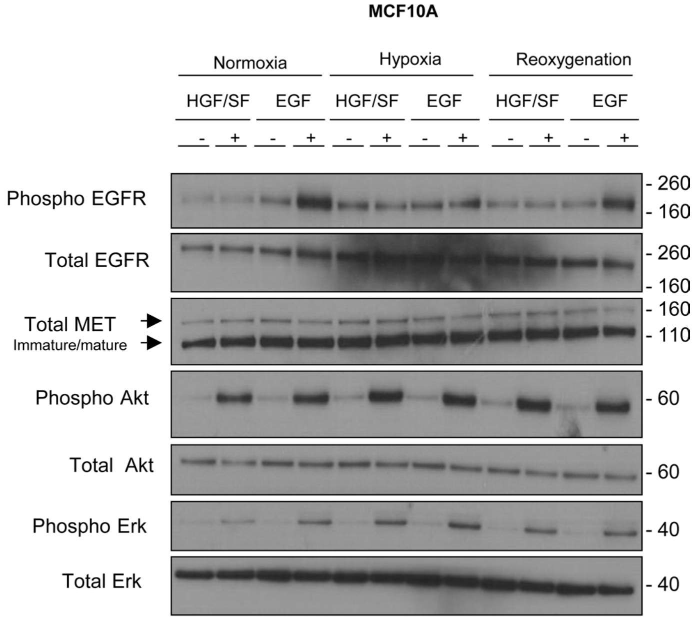

F

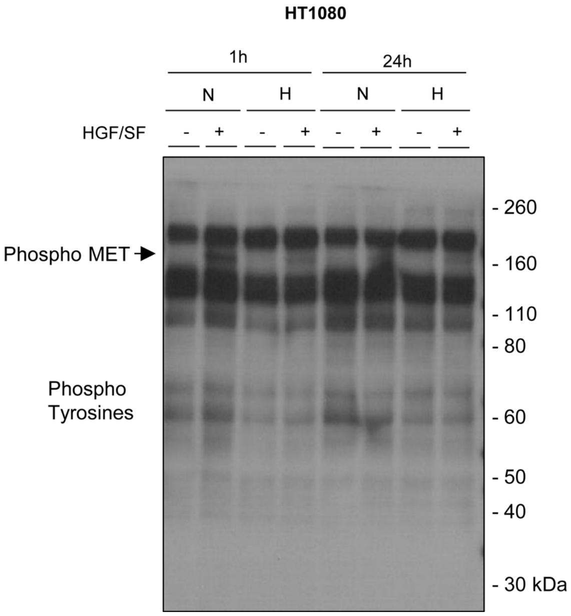

Supplementary Figure 1: Effect of hypoxia on MET phosphorylation and activation of downstream pathways. HT1080 cells (**A**, **F**), HepG2 cells (**B**) and keratinocytes (**C**) were placed under normoxic (N) or hypoxic (H) conditions for 1 or 24 hours and treated or not for 10 minutes with 10 ng/mL HGF/SF. MCF10A cells (**D**) were places for 24 hours and treated or not for 10 minutes with 10 ng/mL HGF/SF. MCF10A cells (**E**) were placed for 1 or 24 hours and treated or not for 10 minutes with 10 ng/mL EGF or 10 ng/mL of HGF/SF. For each cell line, the same amount of protein was resolved by 4–12% Bis-Tris SDS-PAGE and analyzed by western blotting with antibodies directed against one of the following: phosphorylated residues in the MET kinase domain, the MET kinase domain, phosphorylated EGF receptor, EGF receptor, phospho-tyrosines, phosphorylated Akt, Akt, phosphorylated Erk, Erk2, phosphorylated-tyrosines, hypoxia marker HIF1a. The positions of prestained molecular weight markers are indicated. Arrows indicate the positions of precursor and mature full-length MET and Erk1/2 proteins. For Supplementary Figure 1D, the western blot was captured with a CCD camera and levels of the phosphorylated form of MET were quantified. Each level is expressed as a percentage of the level measured under normoxia (n = 3; ± SD).

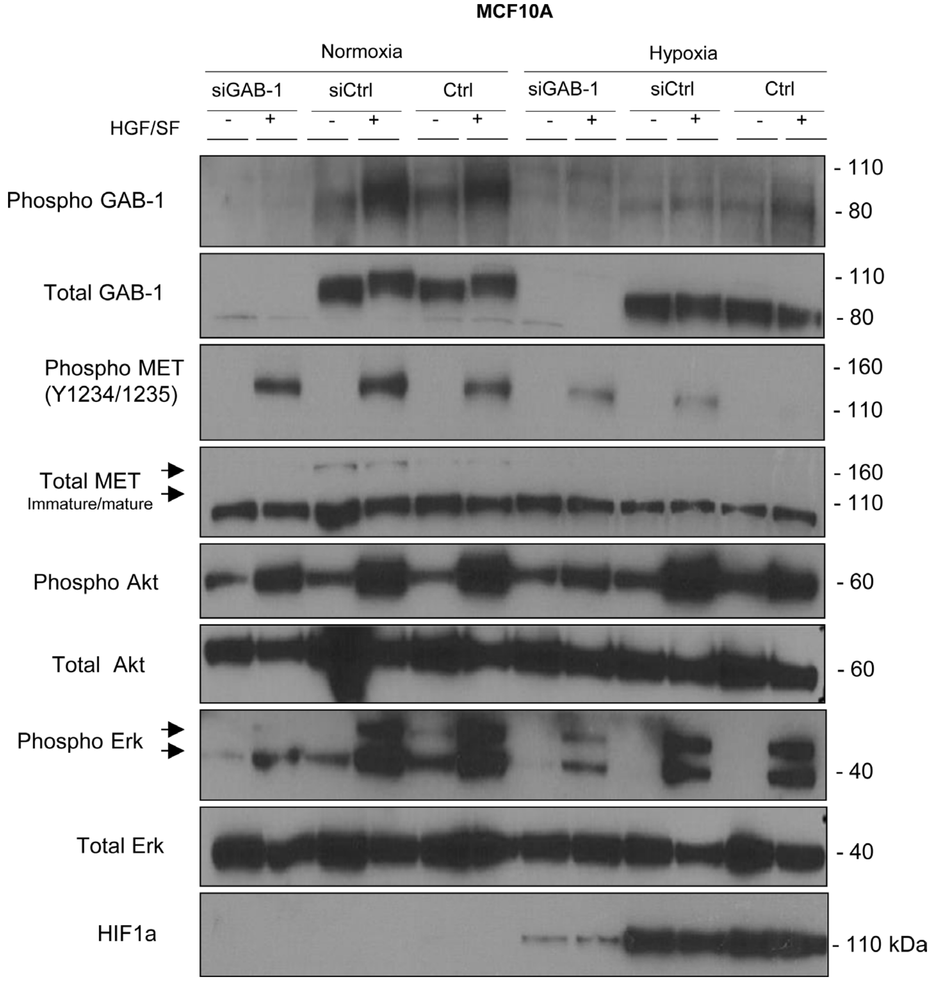

Supplementary Figure 2: Involvement of GAB1 in Akt and Erk pathway activation under hypoxia. MCF10A cells were transfected with a pool of two GAB1-targeting siRNAs, or a control siRNA (siCtrl). A control without siRNA was also included (Ctrl). The cells were then placed for 1 h under normoxic or hypoxic conditions and treated or not for 10 min with 10 ng/mL HGF/SF. In each experiment, the same amount of protein was resolved by 4–12% Bis-Tris SDS-PAGE and analyzed by western blotting with antibodies directed against one of the following: phosphorylated residues in the MET kinase domain, the MET kinase domain, phosphorylated Akt, Akt, phosphorylated Erk, Erk2, phosphorylated GAB1, GAB1, or hypoxia marker HIF1a. The positions of prestained molecular weight markers are indicated. Arrows indicate the positions of precursor and mature full-length MET and Erk1/2 proteins.

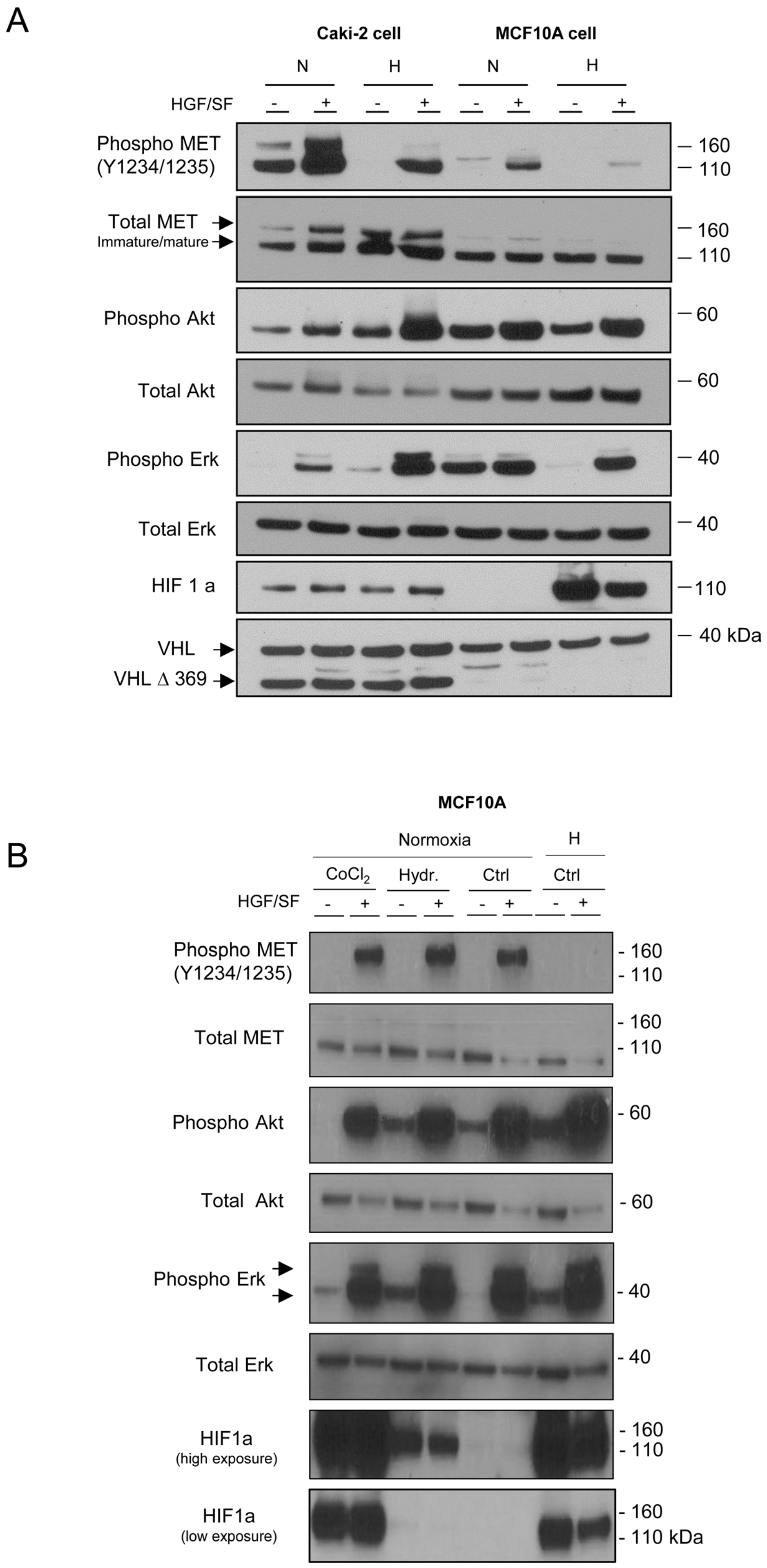

Supplementary Figure 3: Involvement of HIF1a stability in MET phosphorylation. (**A**) Caki2 and MCF10A cells were placed under normoxic (N) or hypoxic (H) conditions for 1h and treated or not for 10 minutes with 10 ng/mL HGF/SF. (**B**) MCF10A cells were treated with $CoCl_2$ (100 μM) or hydralazine (200 μM) and placed under normoxic or hypoxic (H) conditions overnight. They were then treated or not for 10 minutes with HGF/SF at 10 ng/mL. (A, B) The same amount of protein was resolved by 4–12% Bis-Tris SDS-PAGE and analyzed by western blotting with antibodies directed against one of the following: phosphorylated residues in the MET kinase domain, the MET kinase domain, phosphorylated Akt, Akt, phosphorylated Erk, Erk2, hypoxia marker HIF1a or VHL. The positions of prestained molecular weight markers are indicated. Arrows indicate the positions of precursor and mature full-length MET and Erk1/2 proteins.

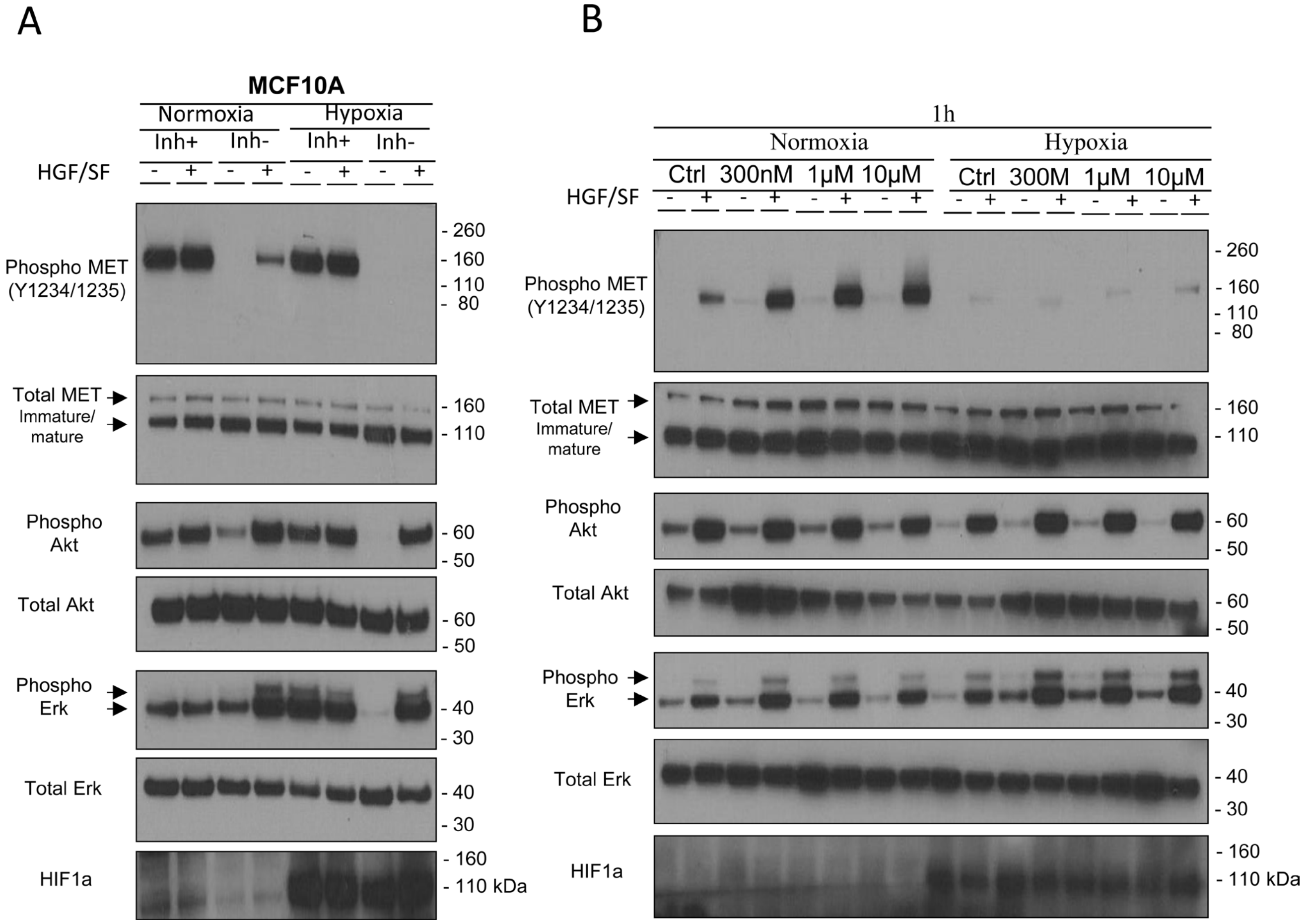

Supplementary Figure 4: A general phosphatase inhibitor restores MET phosphorylation but a Shp1/2-specific phosphatase inhibitor does not. MCF10A cells (**A**, **B**) were placed under normoxic or hypoxic conditions for 1 hour. They were treated or not with sodium orthovanadate (A) or with a Shp-1/2 inhibitor at increasing concentrations (300 nM, 1 µM, 10 µM) (B) for 10 minutes in the presence or absence of HGF/SF at 10 ng/mL. In each experiment, the same amount of protein was resolved by 4–12% Bis-Tris SDS-PAGE and analyzed by western blotting with antibodies directed against phosphorylated residues of the MET kinase domain, the MET kinase domain, phosphorylated AKT, AKT, phosphorylated ERK, ERK2, or hypoxia marker HIF1a. The positions of prestained molecular weight markers are indicated. Arrows indicate the positions of precursor and mature full-length MET and of ERK1/2 proteins.

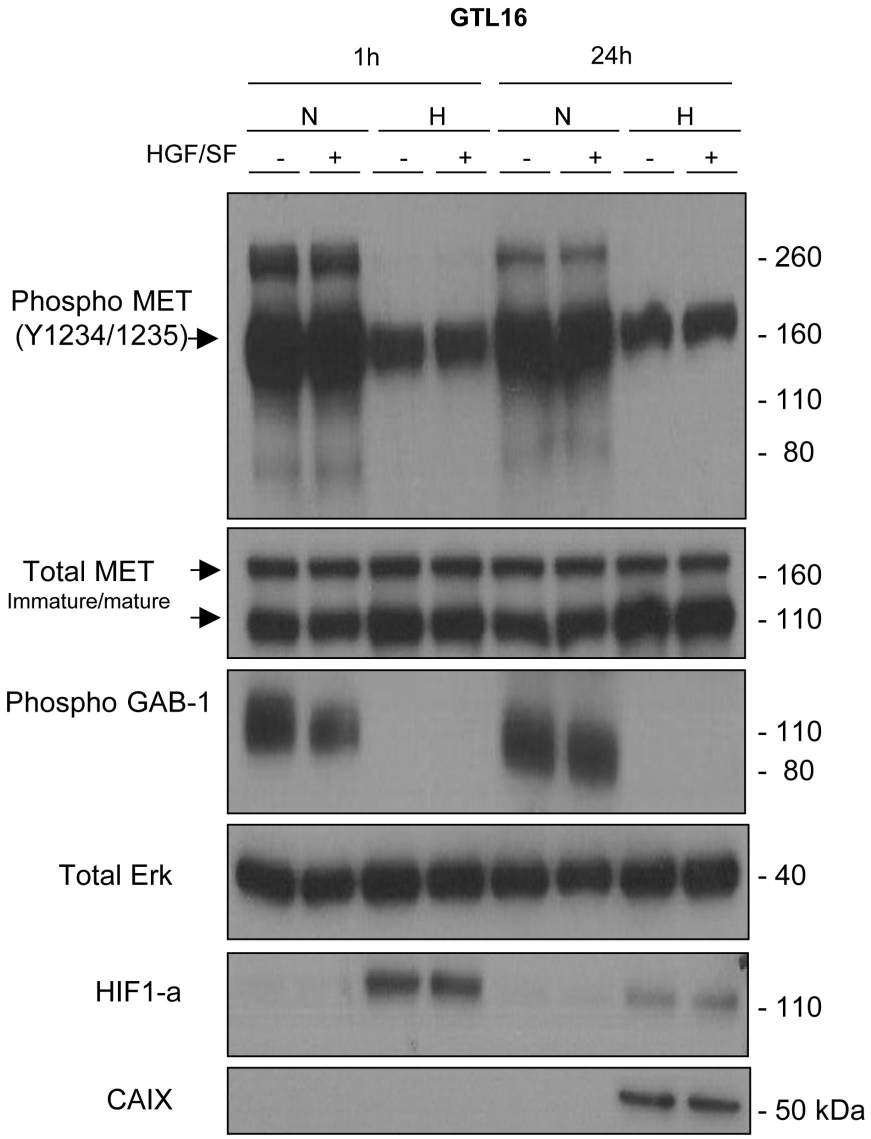

Supplementary Figure 5: Hypoxia causes decreased MET and GAB1 phosphorylation in MET-overexpressing cells. GTL16 cells were placed under normoxic (N) or hypoxic (H) conditions for 1 or 24 h and treated or not for 10 min with 10 ng/mL HGF/SF. The same amount of protein was resolved by 4–12% Bis-Tris SDS-PAGE and analyzed by western blotting with antibodies directed against phosphorylated residues of the MET kinase domain, the MET kinase domain, the hypoxia marker HIF1a or carbonic anhydrase IX, Erk2 and phosphorylated GAB1. The positions of prestained molecular weight markers are indicated. Arrows indicate the positions of precursor and mature full-length MET.

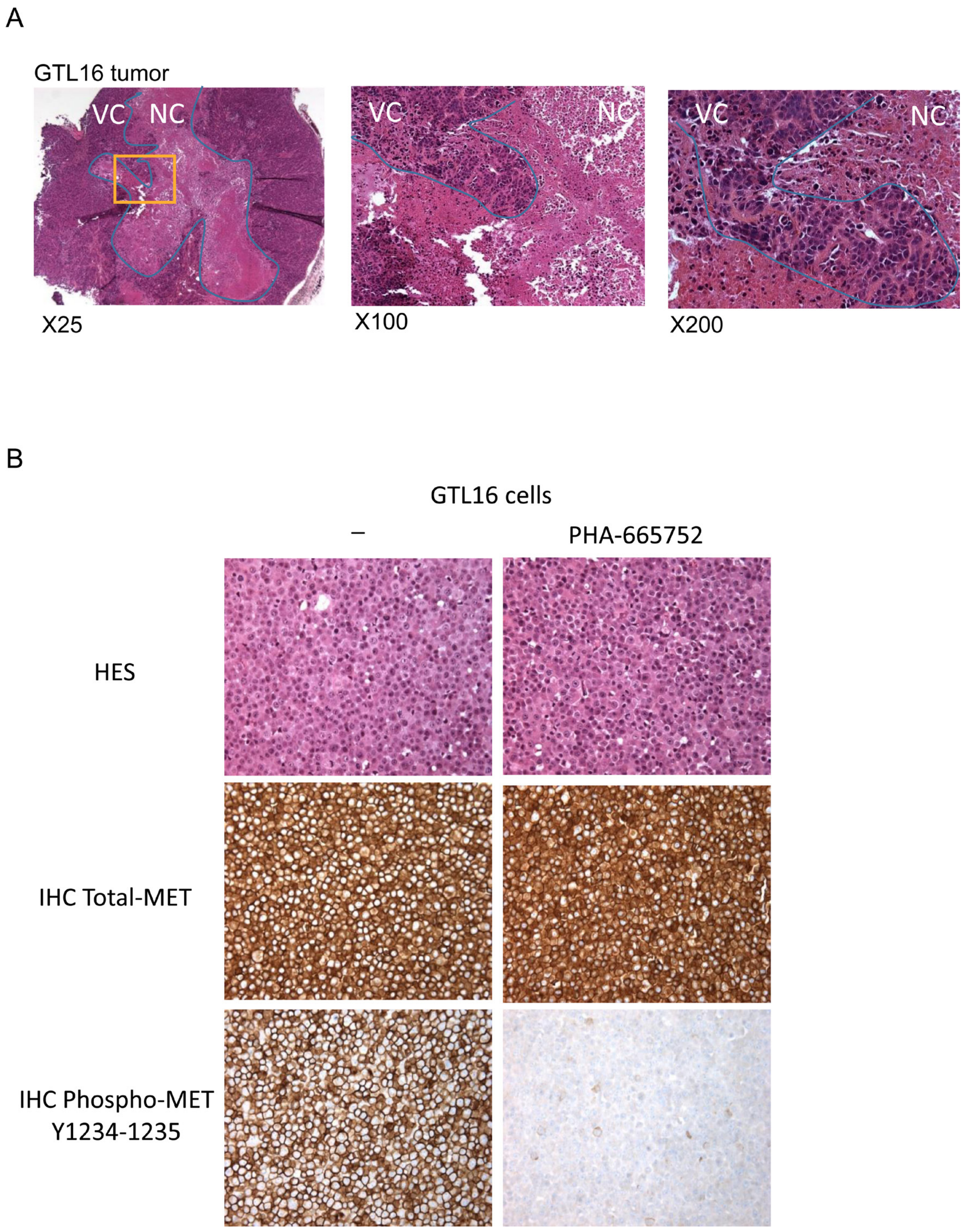

Supplementary Figure 6: Decreased MET phosphorylation in hypoxic areas of GTL16 tumor xenografts. (**A**) GTL16 cells were xenografted subcutaneously into flanks of SCID mice. Seven-µm-thick sections of the tumors were stained with haematoxylin and eosin. left panel: magnification factor ×25; Middle panel: magnification factor ×100; right panel: magnification factor ×200 (applied to the delimited region). NC: Necrotic tumor cells. VC: Viable tumor cells. (**B**) GTL16 cells were treated or not 1 h with 0.3 µM of MET kinase inhibitor PHA-665752. Pellet of the cells were paraffin-embedded (FFPE), stained with haematoxylin and eosin (HES) or IHC was performed with antibodies directed against the intracellular domain of MET (IHC Total-MET) or the phosphorylated form of MET (IHC Phospho-MET Y1234-1235).

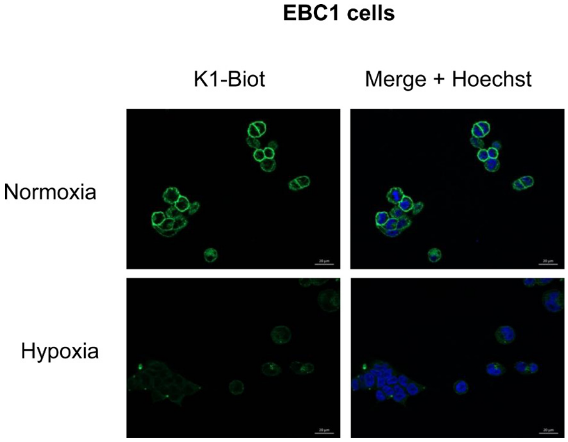

Supplementary Figure 7: Interaction of K1 subdomain of HGF/SF with MET receptor in EBC1 cells. EBC1 Cells were placed under normoxic or hypoxic conditions for 2 h. Cells were incubated 10 min with a complex K1-biotinylated/streptavidin-DyLight 488 (100 nM/50 nM respectively) (green staining). Cells were then fixed and their nucleus stained with Hoechst (blue staining). An overlay of the stains is shown (merge) (scale bar = 20 µm).

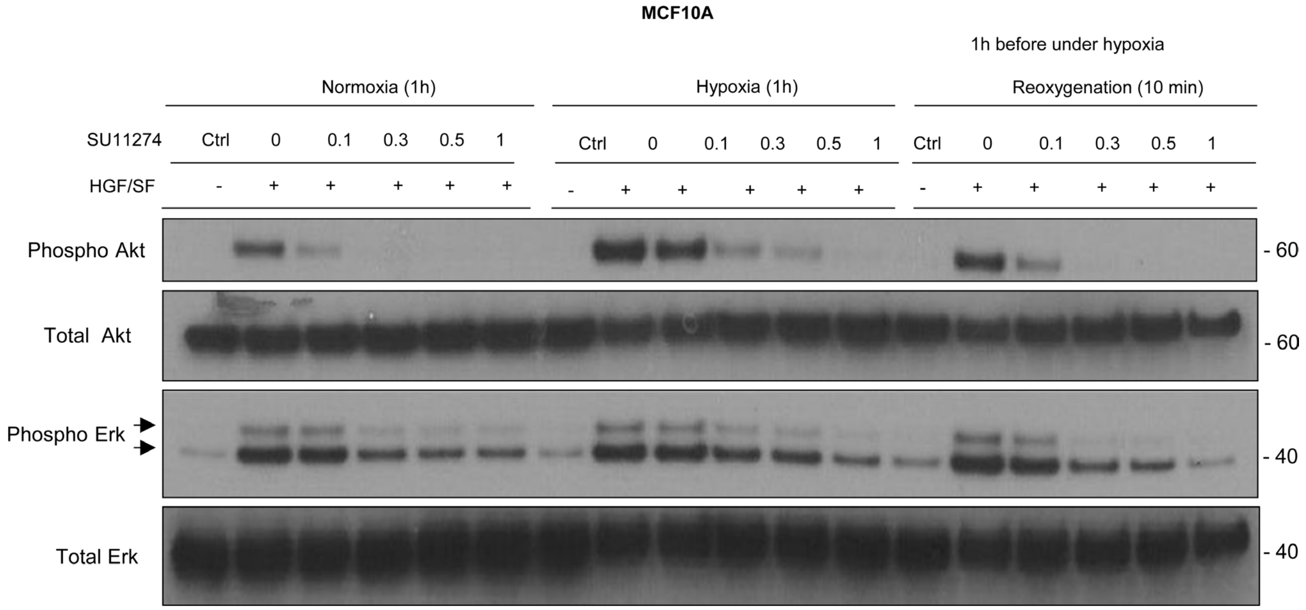

Supplementary Figure 8: Responses to the tyrosine kinase inhibitor SU11274 under hypoxia and reoxygenation. MCF10A cells were treated with the MET tyrosine kinase inhibitor SU11274 at the indicated concentrations and then placed under normoxia or hypoxia for 1 h or under hypoxia for 1 h and then reoxygenated for 10 minutes. The cells were then stimulated or not for 10 min with 10 ng/mL HGF/SF. In all experiments the same amount of protein was resolved by 4–12% Bis-Tris SDS-PAGE and analyzed by western blotting with antibodies directed against phosphorylated Akt, Akt, phosphorylated Erk, or Erk2. The positions of prestained molecular weight markers are indicated. Arrows indicate the positions of Erk1/2 proteins.

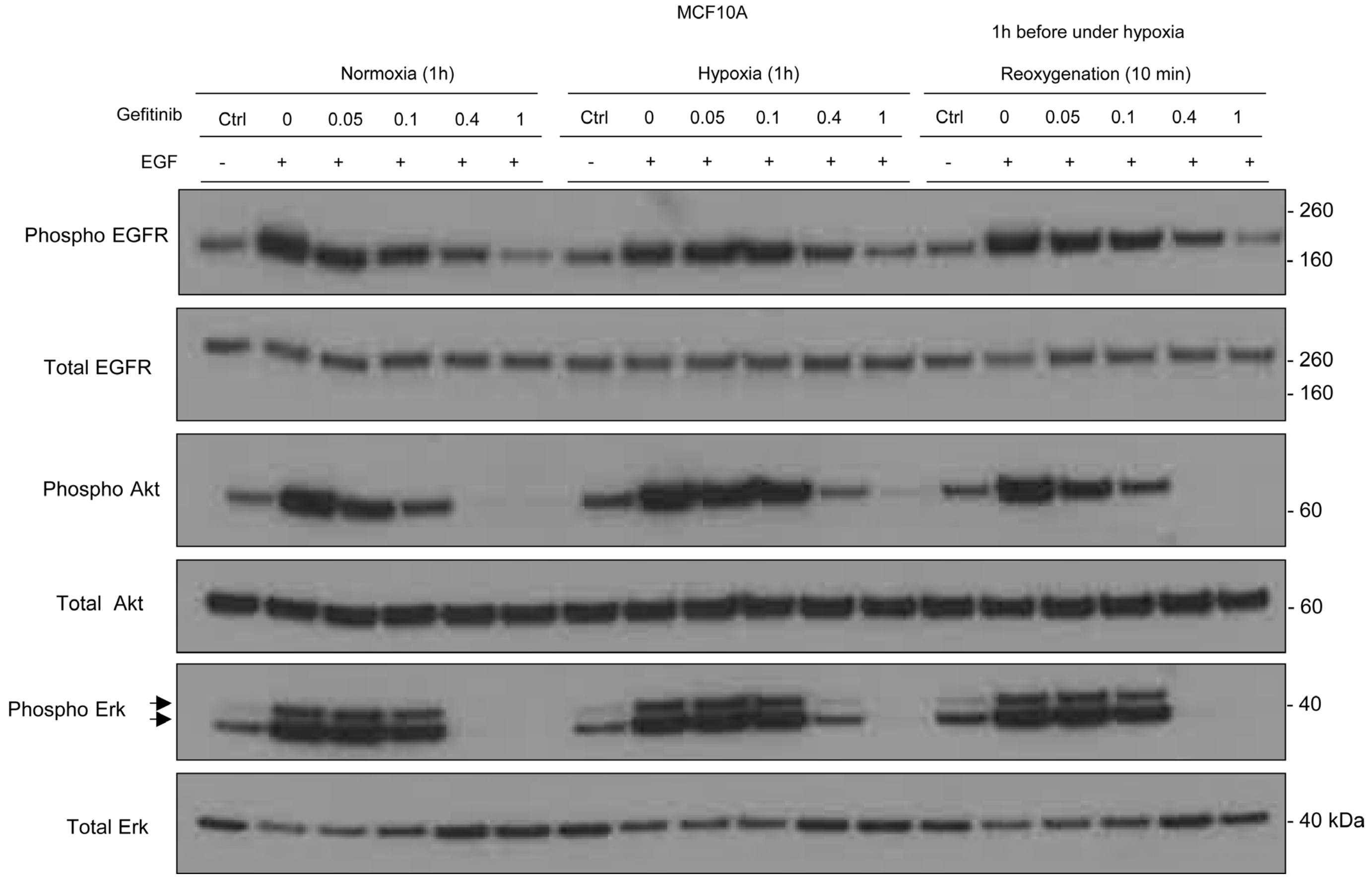

Supplementary Figure 9: Responses to the tyrosine kinase inhibitor gefitinib under hypoxia and reoxygenation. MCF10A cells were treated with the EGFR tyrosine kinase inhibitor gefitinib at the indicated concentrations and then placed under normoxia or hypoxia for 1 h or under hypoxia for 1 h and then reoxygenated for 10 minutes. The cells were then stimulated or not for 10 min with 10 ng/mL EGF. In all experiments the same amount of protein was resolved by 4–12% Bis-Tris SDS-PAGE and analyzed by western blotting with antibodies directed against phosphorylated EGF receptor, EGF receptor, phosphorylated Akt, Akt, phosphorylated Erk, or Erk2. The positions of prestained molecular weight markers are indicated. Arrows indicate the positions of Erk1/2 proteins.
